# Conformational Trajectory of the Molecular Chameleon Grazoprevir From Formulation to Target‐Bound

**DOI:** 10.1002/chem.202502256

**Published:** 2025-12-18

**Authors:** Lianne H. E. Wieske, Guanhong Bu, Máté Erdélyi, Jan Kihlberg, Tamir Gonen, Emma Rova Danelius

**Affiliations:** ^1^ Department of Chemistry for Life Sciences and the Center of Excellence for the Chemical Mechanisms of Life Uppsala University Uppsala Sweden; ^2^ Department of Chemistry University of California Riverside Riverside California USA; ^3^ Department of Biological Chemistry & Physiology University of California Los Angeles Los Angeles California USA; ^4^ Howard Hughes Medical Institute University of California Los Angeles Los Angeles California USA

**Keywords:** chameleonicity, conformational trajectory, grazoprevir, macrocycle, microED, NMR

## Abstract

Macrocycles represent a promising class of beyond‐rule‐of‐5 (bRo5) therapeutics, capable of targeting proteins traditionally considered undruggable by conventional small molecules. Macrocycles exhibit intrinsic flexibility and often display a “chameleon‐like” ability to adapt to their environment, thereby enhancing their oral bioavailability. Describing their structures and conformational changes is essential for advancing their development in the bRo5 space. In this study, we present the novel solid‐ and solution‐state structures of the macrocycle grazoprevir, determined using microcrystal electron diffraction (MicroED) and nuclear magnetic resonance (NMR) spectroscopy. A low‐energy core conformation was consistently identified throughout the conformational journey, from solid formulation to solvated states and upon complexation with the biological target, while the vinyl cyclopropyl sulfonamide side chain reoriented. The presence of a common core conformation (RMSD up to 0.273 Å) suggests that grazoprevir adopts a partial pre‐organized state, optimizing its suitability for target binding. In apolar environment, mimicking the cell membrane, intramolecular hydrogen bonding promoted the formation of compact conformations with reduced radii of gyration and solvent accessible 3D polar surface area, likely facilitating cell permeability. This work presents a comprehensive conformational trajectory of a macrocyclic chameleon and provides potential insights into understanding and characterizing cell permeability and chameleonicity of similar bRo5 therapeutics.

**Abbreviations**
bRo5: beyond‐rule‐of‐five
CSD: Cambridge structural database
cryo‐EM: cryogenic electron microscopy
FDA: food and drug administration
HCV: hepatitis C virus
IMHB: intramoleculcar hydrogen bond
LLMOD: large‐scale low‐mode
MicroED: microcrystal electron diffraction
NAMFIS: NMR analysis of molecular flexibility in solution
MCMM: Monte Carlo multiple minima
NMR: nuclear magnetic resonance
MMFF: Merck molecular force field
NS: non‐structural
NOE: nuclear Overhauser effect
NOESY: nuclear Overhauser effect spectroscopy
OPLS: optimized potentials for liquids simulations
PANIC: peak amplitude normalization for improved cross‐relaxation
PBF: Poisson‐Boltzmann finite elements
PDB: protein data bank
PRCG: Polak‐Ribiere type conjugate gradient
RMSD: root‐mean‐square deviation
Ro5: rule‐of‐five
SA 3D PSA: solvent accessible 3D polar surface area
TEM: transmission electron microscope
R_gyr_
: radius of gyration

## 1 Introduction

Macrocycles are a class of large and flexible compounds that occupy the chemical space beyond the traditional “rule of 5” (bRo5), a framework used to predict oral bioavailability [1, 2]. Despite falling outside these conventional guidelines, many bRo5 drugs are orally active, suggesting they possess intrinsic conformational flexibility, or “molecular chameleonicity”, that enables them to modulate their polarity in response to different environments [3]. This adaptability allows them to balance aqueous solubility with efficient membrane permeability [4, 5, 6, 7]. In contrast to small molecule drugs, compounds in the bRo5 space have gained prominence in targeting traditionally undruggable targets and diseases [8, 9, 10]. Small‐molecule drugs within the Ro5 framework are typically designed using 2D molecular descriptors including molecular weight, number of hydrogen bond donors, and acceptors, and calculated lipophilicity (cLogP). In the space bRo5 additional descriptors which depend on the conformations adopted by the compounds, such as solvent‐accessible 3D polar surface area (SA 3D PSA), radius of gyration (*R*_gyr_), and intramolecular hydrogen bond (IMHB) count are used to optimize the suitability for oral administration [2, 4, 5, 11, 12]. However, applying these metrics to macrocycles remains challenging due to the limited availability of experimental structural data, which hampers the establishment of reliable design principles for this compound class.

**FIGURE 1:**
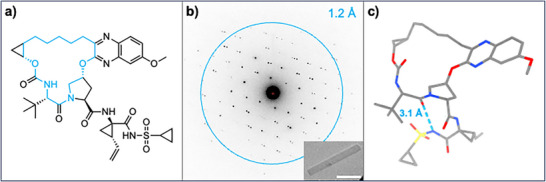
The crystal structure of grazoprevir determined by MicroED. (a) Chemical structure of grazoprevir with the macrocyclic core highlighted in blue. (b) Representative image of the grazoprevir crystal and MicroED pattern. The scale bar corresponds to 3 µm. The resolution is indicated by the blue ring and number. (c) MicroED structure of grazoprevir. Atom colors: C, gray; N, blue; O, red; S, yellow. The intramolecular hydrogen bond is shown as a blue‐dashed line.

Grazoprevir (Figure 1a) is a second‐generation hepatitis C virus (HCV) nonstructural 3/4A (NS3/NS4A) protease inhibitor developed by Merck in 2012 and approved by the FDA in 2016 as part of an orally administered combination therapy with elbasvir [13, 14]. Like most NS3/NS4A protease inhibitors, grazoprevir is orally available despite its limited water solubility (Table S21) [15]. The potential chameleonicity of grazoprevir has not yet been structurally characterized. Determining the structures of bRo5 inhibitors like grazoprevir in various states is crucial for understanding their chameleonic behavior and mechanisms of oral absorption. Yet, due to their intrinsic flexibility, resolving their solid‐ and solution‐state structures remains a significant challenge. Neither single‐crystal nor powder X‐ray structures are available in the Cambridge structural database (CSD) for any macrocyclic NS3/NS4A inhibitor, and are scarcely found for linear derivatives such as asunaprevir [16]. The recent solid‐state structures of the macrocycles simeprevir and paritaprevir, determined by microcrystal electron diffraction (MicroED) [17, 18], marked the first entries of macrocyclic NS3/NS4A inhibitors in the CSD. MicroED is a cryogenic electron microscopy (cryo‐EM) method that uses sub‐micrometer‐sized crystals to determine structures from continuous‐rotation electron diffraction data collected in a cryogenic transmission electron microscope (TEM). This method has proven particularly valuable for solving challenging structures that are not accessible by conventional X‐ray crystallography [19, 20, 21, 22]. For a full understanding of the chameleonic and pharmacological properties of bRo5 inhibitors, the solution‐state and target‐bound conformations also need to be evaluated. In solution, macrocycles in the bRo5 space typically adopt dynamic ensembles of rapidly interconverting conformations and cannot be represented by a single static structure [6, 23, 24]. To elucidate their full ensembles, computational sampling combined with solution‐state nuclear magnetic resonance (NMR) spectroscopy across different environments, is commonly employed [6, 23, 25, 26], for example using data deconvolution by the NMR analysis of molecular flexibility in solution (NAMFIS) algorithm [27, 28]. Thus, combining solid‐ and solution‐state data along all steps of the macrocycles conformational journey provides valuable information for understanding their chameleonic behaviors. For instance, a study of the solid form complexity of macrocycle paritaprevir, where aqueous, membrane‐permeable and target‐bound conformations were assessed computationally [24], suggested that both SA 3D PSA and *R*_gyr_ were elevated in polar environments and in the target‐bound state, but significantly reduced in apolar environments. Specifically, the polar‐to‐apolar span was 76 Å^2^ for SA 3D PSA and 0.36 Å for *R*_gyr_, helping to explain paritaprevir's favorable absorption profile [24].

bRo5 compounds can be full chameleons (roxithromycin, telithromycin, spiramycin), partial chameleons (simeprevir, asunaprevir, atazanavir), or nonchameleons (rifampicin, daclatasvir) [5, 6, 23]. Here we describe the experimental structural elucidation of grazoprevir for all stages of its conformational journey. The solid‐state structure was determined using MicroED, while the solvated and membrane‐permeable conformational ensembles were characterized using NAMFIS. Key 3D molecular descriptors such as SA 3D PSA, *R*_gyr_ and IMHB were calculated for each conformation to assess the degree of chameleonicity. The structural data are contextualized by comparison with available target‐bound structures from the Protein Data Bank (PDB), providing a comprehensive view of grazoprevir's conformational trajectory from formulation to target engagement. The insights gained from this analysis advance our understanding of molecular chameleonicity and contribute to the long‐term goal of rationally designing cell‐permeable drug candidates in the bRo5 chemical space.

## 2 Results and Discussion

### 2.1 Solid‐State Structure

The crystal structure of grazoprevir was solved by MicroED directly from the powder formulation. The grazoprevir powder was applied to a continuous carbon‐coated TEM grid without further sample preparation, and plate‐like microcrystals were identified using low‐magnification whole‐grid atlas in the TEM (Figure 1b). The final structure was determined from the MicroED dataset obtained from a single crystal, collected by continuously rotating the microcrystal within an angular wedge of 138 degrees using a rotation speed of 2 degrees per second, at an electron dose rate of 0.01 e^−^/Å^2^/s. The structure was solved at 1.0 Å resolution in the orthorhombic space group P2_1_2_1_2_1_ and refined to an *R*_1_ value of 0.159 (Table S1). A single conformation was found in the asymmetric unit of the crystal structure (Figure 1c), in which grazoprevir adopts a flat macrocyclic core with the sidechain in a folded conformation due to the twisted proline ring connecting the core and the vinyl cyclopropyl sulfonamide. This conformation along with crystal packing results in an IMHB between the sulfonamide amide and one of the core carbonyl oxygen atoms (Figure 1c). The crystal packing analysis revealed that the packing is primarily driven by hydrophobic interactions (Figure S1). The solvation energy of the crystal structure was calculated to −31.3 kcal/mol using standard DFT methods at a level of theory suitable for routine organic compounds (containing C, H, N, O, and S atoms), and the SA 3D PSA and *R*_gyr_ were determined to be 174 Å^2^ and 5.57 Å, respectively (Figure 4 and Table S10).

### 2.2 Solution Ensembles

The behavior of grazoprevir in solution was determined using nuclear Overhauser effect (NOE) NMR experiments. Flexible molecules, such as grazoprevir, show a single set of NMR signals that does not originate from a single conformation, but from a set of rapidly interconverting geometries. To accurately describe the full conformational ensembles, the NMR data was deconvoluted using the NAMFIS algorithm [27, 28]. The analysis was performed in polar (DMSO‐*d_6_*) and apolar (CDCl_3_) environments, mimicking the extracellular and membrane environments, respectively [6, 29, 30, 31].

**FIGURE 2:**
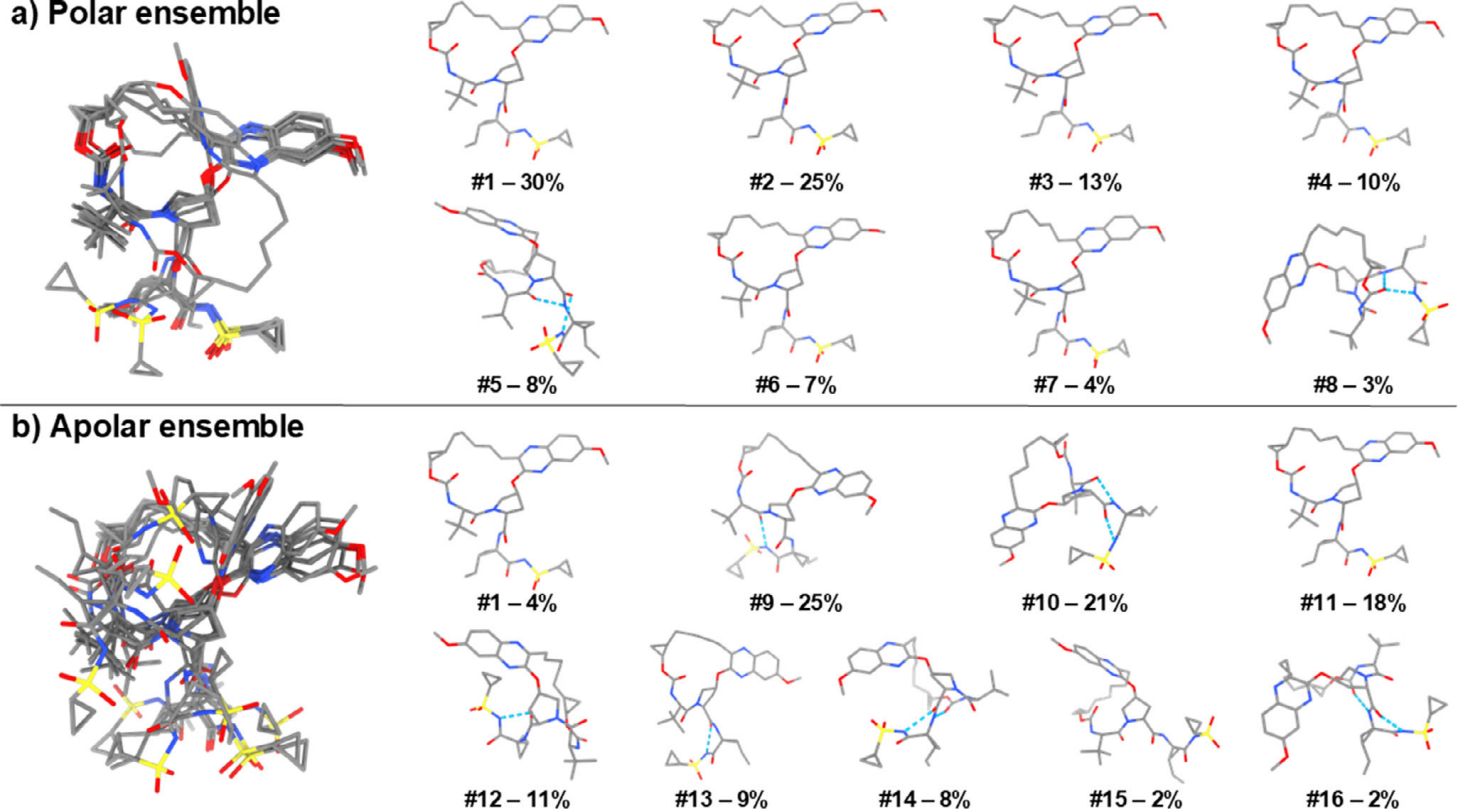
The solution ensembles of grazoprevir elucidated by NAMFIS. All solution conformations in (a) polar environment (DMSO‐d_6_; mimicking the extracellular space), and (b) apolar environment (CDCl_3_; mimicking the cell membrane), superimposed using the macrocyclic core. The individual conformations displaying intramolecular hydrogen bonds indicated with blue dotted lines are displayed with their corresponding population %. Hydrogen bond lengths are given in Figures S5 and S6.

In polar environment, the solution ensemble was found to consists of eight conformations with populations between 3% and 30% (Figures 2a, S5 and Table S7). The majority of the polar ensemble (89%, conformations 1–4, 6 and 7) shares a similar flat macrocyclic core conformation with the solid‐state structure (RMSD between 0.153 and 0.505, Table S13) but adopts more open geometries, lacking the folded side‐chain conformation and the IMHB observed in the solid state. Yet, the ensemble revealed an overall flexibility in the polar solvated state, as illustrated by the root‐mean‐square deviation (RMSD) values for all heavy atoms ranging from 0.319 to 3.898 Å across the different conformations (Table S11). The calculated solvation energy of the conformations varied between −42.1 and −25.2 kcal/mol with a population‐weighted average of −37.5 kcal/mol (Figure 4 and Table S10), which is 6.2 kcal/mol lower than the solid state, suggesting that breaking of the IMHB and dissolution into polar environment from the solid state is favorable. Indeed, the greatest flexibility was observed in the vinyl cyclopropyl sulfonamide moiety as well as in the alkyl chain of the macrocyclic core. Four new IMHBs were identified in the polar environment, corresponding to 11% of the solution ensemble (conformations 5 and 8, Figures 2a and S5). The SA 3D PSA of the polar conformations varied between 144 and 198 Å^2^ (Figure S13 and Table S10) with a span of 54 Å^2^ and a population‐weighted average of 185 Å^2^. The *R*_gyr_ of the polar conformations ranged from 5.02 to 5.78 Å, with a population‐weighted average of 5.64 Å (Figure S13 and Table S10). The two conformers exhibiting IMHBs showed the smallest *R*_gyr_ values, 5.25 Å and 5.02 Å, with corresponding SA 3D PSA values of 144 Å^2^ and 172 Å^2^ for conformations 5 and 8, respectively.

The solution ensemble in apolar environment consists of nine conformations with populations between 2 and 25% (Figure 2b, Figure S6 and Table S7). Interestingly, the major conformation in apolar environment (conformation 9, Figure 2b, 25%) is the same as the solid state MicroED structure. The apolar ensemble showed a slightly larger overall degree of variation than the polar ensemble with an RMSD span for all heavy atoms between 0.427 and 4.354 Å (Table S11), although 47% (conformations 1, 9 and 11) share the overall geometry with the solid‐state structure. The solvation energies varied from −28.4 to −18.7 kcal/mol with a population‐weighted average of −24.1 kcal/mol (Figure 4 and Table S10). A significantly larger portion of the apolar ensemble contained IMHBs; nine IMHBs covering 76% (conformations 9, 10, 12, 13, 14 and 16), of which seven were shared with the polar ensemble and two were unique to the apolar one (Figure 2b and S6). The *R*_gyr_ of the apolar ensemble varied between 4.92 and 5.75 Å (Figure S13 and Table S10) with a population‐weighted average of 5.36 Å, which is significantly lower than in the polar environment. The SA 3D PSA spanned 79 Å^2^ with values between 131 and 210 Å^2^ (Figure S13 and Table S10), with a population‐weighted average of 180 Å^2^, which is slightly lower than for the polar ensemble.

### 2.3 Target‐Bound Structures

**FIGURE 3:**
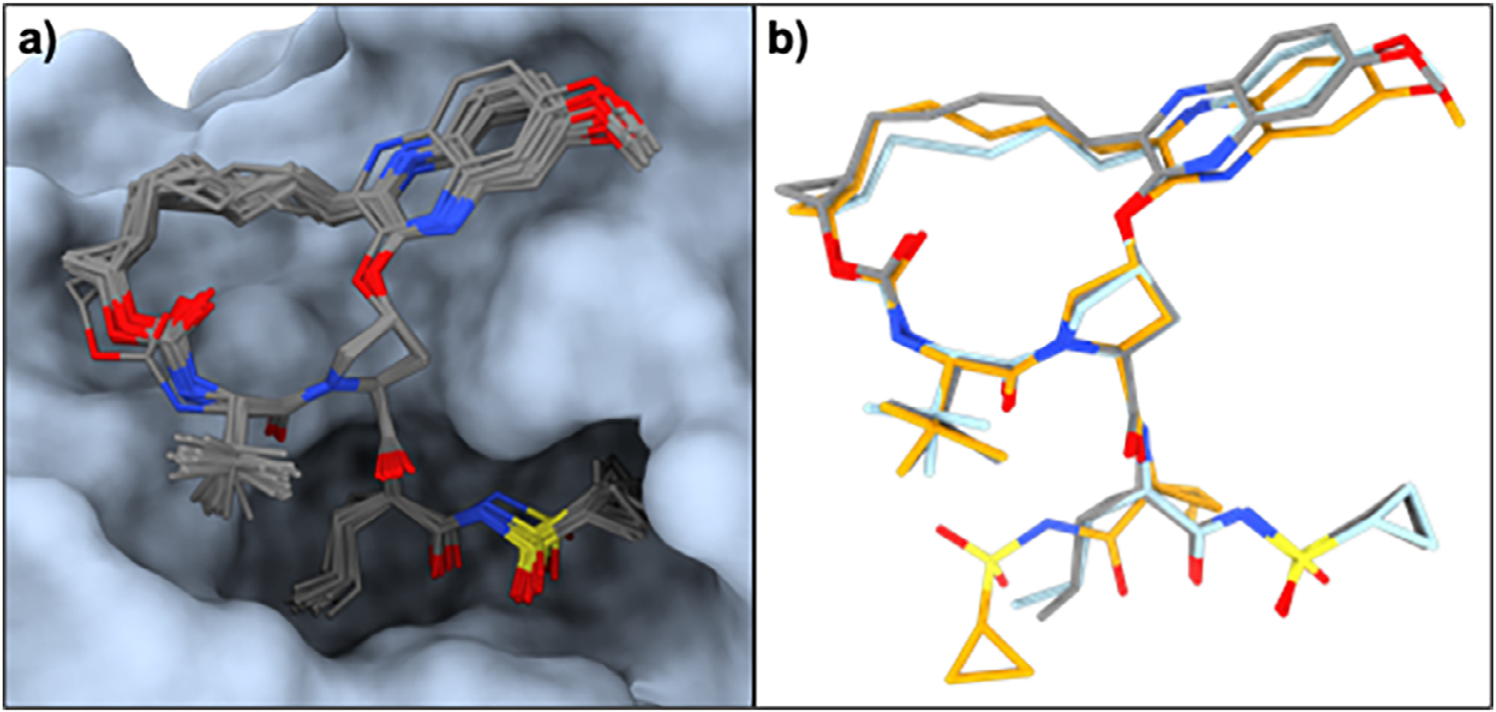
(a) The 19 target‐bound conformations of grazoprevir obtained from the six PDB structures (IDs: 3SUD, 3SUE, 3SUF, 3SUG, 6C2M, and 6P6Q) show conformational flexibility in the alkyl chain of the macrocyclic core, and in some of the side chains. (b) Superimposition of the solid state MicroED structure (orange), which is identical to the most populated apolar conformation (#9, 25%), with the most populated polar solution conformation (#1, 30%, gray), and the target‐bound structure determined in PDB ID 3SUE chain K (cyan). All share a similar macrocyclic core, while the two solid‐state conformations differ in the sidechain orientation.

**FIGURE 4:**
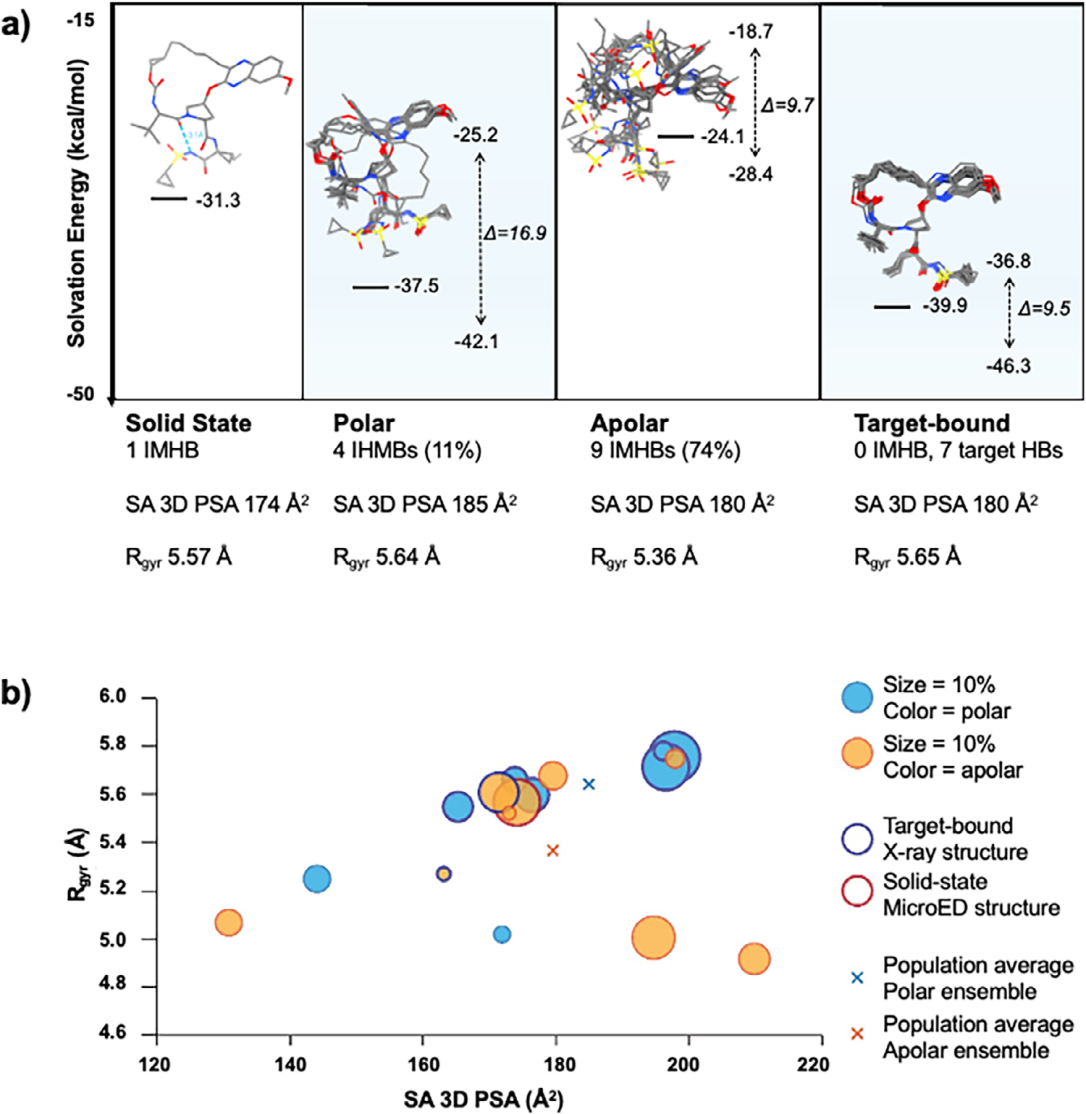
(a) Proposed conformational journey of grazoprevir from its drug formulation, its dissolution in solutions (polar and apolar environment), to its destination on protein targets. The different panels show the experimental structures as determined in the solid state (MicroED), polar solution (NMR, DMSO‐d_*6*_), apolar solution (NMR, CDCl_3_), and bound to the biological target HCV NS3/NS4A (X‐ray crystallography) [32]. The population‐weighted average solvation energies are shown as well as the highest and lowest energy values next to the dashed lines. The hydrogen bond count, the population‐weighted average solvent accessible 3D polar surface area (SA 3D PSA) and the population‐weighted average radius of gyration (*R*_gyr_) are shown under each plot for comparison. (b) The *R*_gyr_ of the solution conformations plotted as a function of their corresponding SA 3D PSA with conformations found in polar environment given in blue and apolar in orange. The size of each circle corresponds to its population, with a 10% reference size in the legend. Selected target‐bound and MicroED structures are highlighted with a thicker border (in blue for target‐bound and red for MicroED). The population‐averaged *R*_gyr_ and SA 3D PSA for the polar (blue) and apolar (orange) ensembles are marked with a cross.

In the target bound state (Figure 3a) [32] grazoprevir occupies the same binding pocket as other HCV protease inhibitors and adopts a unique conformation that stacks against the catalytic triad (H57, D81 and S139). This conformation has been associated with increased resistance to common mutations, such as R155K and S168A [32]. Key interactions include a hydrogen bond between the sulfonamide moiety and S139 as well as stacking between H57 and D81 and the quinoxaline moiety (Figure S12) [32]. Six crystal structures of grazoprevir in complex with its target have been deposited into the PDB, containing one, two or four chains in the asymmetric unit, resulting in a total of 19 target‐bound conformations (Figure 3a and Table S10) [32]. These conformations consistently display a flat macrocyclic core, closely resembling the one of the solid‐state MicroED conformation (RMSD between 0.153 and 0.273, Table S13), offering an optimal fit within the HCV protease active site. Although the structural deviations among the target‐bound conformers are relatively small, with RMSD values ranging from 0.207 to 0.552 Å (Table S12), it is important to emphasize that the target‐bound state of this flexible macrocycle is best represented as an ensemble of conformations, rather than a single static structure (Figure 3a). The most notable variations occur in the vinyl cyclopropyl sulfonamide side chain and the methylene linker of the macrocyclic core (Tables S12, S14, S16, S18, S20). Solvation energies for the target‐bound conformations range from –46.3 to –36.8 kcal/mol, with an average of –39.9 kcal/mol (Figure 4 and Table S10), which is lower than those of other experimental states, suggesting that target binding is energetically favorable. The *R*_gyr_ remains relatively consistent across the ensemble (5.54–5.85 Å), while the SA 3D PSA spans a broader range (165–205 Å^2^), but staying within the range observed in both solvent‐state ensembles (Figure 4, Table S10).

### 2.4 Structural Comparison

The solid‐state MicroED structure was identified as the most abundant conformation (25%) in the apolar environment. Although the side‐chain orientation of this structure is only sparsely populated in the polar environment, the macrocyclic core conformation observed in the solid‐state structure was present in 89% (conformations 1–4, 6 and 7) of the polar ensemble, as compared to 47% (conformations 1, 9 and 11) of the apolar ensemble (Tables S7 and S13). Similarly, the solid‐state structure shares its core conformation with the target‐bound structures (core RMSD values between 0.153 and 0.505 Å; Supporting information Table S13) but differs in the orientation of the vinyl cyclopropyl sulfonamide side chain (Figure 3b). Conformations identical to eight target‐bound structures were identified within the solution ensembles, comprising 89% (conformations 1–4, 6 and 7) of the polar and 22% (conformations 1 and 11) of the apolar ensemble. These findings suggest that, for grazoprevir, the solid‐state formulation closely resembles the cell‐permeable state, while the polar solution ensemble is highly representative of the target‐bound conformation, although sharing the same core geometry (Figure 3b). The solvent‐accessible SA 3D PSA of the MicroED structure is 174 Å^2^, which is found to be a bit lower than the population‐weighted averages of the NMR ensembles (185 and 180 Å^2^ in polar and apolar environments, respectively) and the average SA 3D PSA of the target‐bound structures (180 Å^2^, Figures 4 and S13). Even though the difference is small, the PSA represent the conformational changes in the different environments. Similarly, The *R*_gyr_ is 5.57 Å for the solid‐state structure, which lies between the population‐weighted averages of the two solution ensembles—5.64 Å in the polar and 5.36 Å in the apolar environment. Notably, the average *R*_gyr_ of the target‐bound structures (5.65 Å) closely matches that of the polar ensemble (Figures 4 and S13). Hence, both PSA and *R*_gyr_ can be concluded to report on the conformational changes of grazoprevir in different environments.

### 2.5 Molecular Chameleonicity

For bRo5‐drugs, 3D descriptors such as the *R*_gyr_ as a measure of size instead of molecular weight, and the SA 3D PSA instead of the number of hydrogen bond donors and acceptors, often provide a better view to explain their aqueous solubility and membrane permeability [4, 33]. Even though the exact values of the *R*_gyr_ and SA 3D PSA for a single conformation might not be informative, they can give a perspective when compared for conformations between solvents or between similar compounds [6, 23]. In solution the SA 3D PSA varies by 79 Å^2^ (Supporting information Figure S13) between the conformations of grazoprevir from both environments, with the solid‐state structure and most of the target‐bound structure having PSA values within the range of the solution ensemble. This range is very similar to the variation found for other HCV inhibitors such as asunaprevir (88 Å^2^) [6], paritaprevir (76 Å^2^) [24], and simeprevir (72 Å^2^) [6]. The spread in *R*_gyr_ for the solution ensembles of grazoprevir (0.86 Å, Figure S13) is below the ones reported for asunaprevir (1.22 Å) and simeprevir (1.11Å) [6], but above the reported *R*_gyr_ range for the solution conformations of paritaprevir (0.36 Å) [24]. The population‐weighted average *R*_gyr_ in polar environment (5.64 Å) is larger than in apolar environment (5.36 Å). In addition, grazoprevir shows a large difference in IMHB between polar and apolar environments. All IMHBs involve the vinyl cyclopropyl sulfonamide side‐chain, but IMHBs are only found for 11% (conformations 5 and 8) in polar environment as compared 76% (conformations 9, 10, 12, 13, 14, and 16) in apolar environment (Figure 2). Thus, in line with simeprevir and asunaprevir, the chameleonic behavior of grazoprevir is well‐reflected in IMHB patterns, PSA and *R*_gyr_ values (Figures 4 and S13) determined for the two solution ensembles, whereas the overall flexibility is best reflected by the RMSD values. In the target‐bound states, grazoprevir shows *R*_gyr_ variation of 0.31 Å and SA 3D PSA span of 40 Å^2^ (Figure S13), suggesting its ability to adjust the appearance to accommodate multiple genotypes and mutants and retain potency.

### 2.6 Conformational Trajectory

Grazoprevir has a folded side‐chain conformation in the solid‐state, with an IMHB from the sulfonamide to the macrocyclic core (Figure 1c), and crystal packing driven by intermolecular hydrophobic interactions. Once dissolved, grazoprevir populates a large conformational space (Figures 2a and supporting information Figures S5,6), as is indicated by the dissimilarity between its solution conformations (RMSD values between 0.313 and 4.354 Å). One conformation is shared by the polar and apolar states; the predominant conformation in the polar phase (conformation 1, Figure 2a,b) is present at low abundance in the apolar phase, suggesting it serves as a transitional conformation between the two states [29, 34]. This conformation also shares the core conformation with the solid‐state structure as well as the target‐bound structure. The conformational changes required for membrane permeability are driven by the formation of multiple IMHBs, increasing the hydrogen bonded population from 11% to 76%, resulting in presumably cell‐permeable conformations characterized by a mild reduction in SA 3D PSA as well as *R*_gyr_. Hence, conformations with low PSA and *R*_gyr_ could be the reason for the high permeability of grazoprevir. For example, conformation #14 (Figure 2b) found in apolar environment has a low *R*_gyr_ (5.07 Å), a low PSA (131 Å^2^) and two IMHBs. Thus, this conformation may promote permeability and could serve as a template for optimization. In the final step of the journey, grazoprevir adopts several different conformations when bound to variants of the NS3/NS4A protease, while maintaining the macrocyclic core similar to the solid‐state structure, the majority of polar conformation and the most abundant apolar conformation (Figure 3b). The vinyl cyclopropyl sulfonamide side‐chain forms an IMHB in the solid‐state and apolar environment, important for crystal packing as well as membrane permeability; and is reoriented 180° to break IMHB and expose polar moieties, important for aqueous solubility as well as binding to the target with several intermolecular hydrogen bonds (Figures 3b and S12).

These structural analyses clearly indicate that the flat conformation of grazoprevir's macrocyclic core represents a low‐energy state, as it is consistently observed across all three methods (Figure 3 and Supporting Information Table S10) and ranks among the most populated conformations in both polar and apolar solution environments. Hence, the cyclization of the linear precursor to form this flat macrocyclic core effectively pre‐organizes grazoprevir into a bioactive, low‐energy conformation. Despite this preorganization, grazoprevir retains substantial conformational flexibility in particular for the vinyl cyclopropyl sulfonamide side chain, which is crucial for both membrane permeability and effective target binding—particularly given the dynamic nature of the HCV protease, where regions of the binding site are poorly defined due to intrinsic flexibility. The dynamic adaptability can also be speculated to increase resistance to amino acid mutations. Notably, the orientation of the aromatic moiety and its attached flexible alkyl chain exhibits significant variability, corresponding to the least well‐defined areas of the binding pocket (Figure 3a). This flexibility supports an induced‐fit binding mechanism, which is essential for engaging such a dynamic target as the HCV protease [35]. The energy profile of grazoprevir along its conformational trajectory (Figure 4) suggests, as anticipated, that dissolution from the solid state into a polar environment is energetically favorable, while membrane permeation seem to present a slight energy barrier. Target binding, however, is estimated to be the most energetically favorable state, exhibiting the lowest calculated energy among all conformational states.

## 3 Conclusions

This study presents a comprehensive analysis of a macrocyclic drug's conformational journey by experimental means; from the solid state, through solution environments, to the target‐bound state. The novel crystal structure of grazoprevir was determined directly from powder material using MicroED, overcoming previous challenges likely caused by the compound's inherent flexibility and crystallization difficulties. By determining solution‐state ensembles of grazoprevir in both polar and apolar environments—mimicking extracellular and membrane‐like conditions—and comparing these to previously reported target‐bound structures, we completed the full conformational trajectory. Across all states, a flat macrocyclic core conformation representing a low‐energy state was consistently identified, suggesting that cyclization of the linear precursor effectively pre‐organizes the core into its bioactive form. In solution, grazoprevir undergoes multiple conformational changes, and its substantial flexibility appears essential for both membrane permeability and target engagement. This dynamic behavior is driven by the formation and disruption of IMHBs, which result in conformations with varying SA 3D PSA and *R*_gyr_. Our findings suggest that the solid‐state formulation closely resembles the cell‐permeable state, while the polar solution ensemble closely mirrors the bioactive, target‐bound conformation. The highly flexible vinyl cyclopropyl sulfonamide side chain plays a critical role in membrane permeability, forming stabilizing hydrogen bonds in apolar environments that effectively reduce the overall polarity (SA 3D PSA) and molecular size (*R*_gyr_), thereby enabling passive diffusion across the membrane. Altogether, the conformational journey of grazoprevir presented here provides valuable insights for optimizing solid‐state formulations, enhancing membrane permeability, and improving target binding of similar macrocyclic bRo5 inhibitors. Much like asunaprevir and simeprevir, grazoprevir does not show a distinct chemical space being occupied in polar as compared to apolar environment. Instead, it shows a gradient from apolar to polar environments, the space of which covers the MicroED as well as the target‐bound state. The population‐averaged SA 3D PSA and *R*_gyr_ are slightly lower for the apolar ensemble as compared to the polar ensemble, allowing us to classify it as a partial molecular chameleon [6]. Despite similar changes in 3D descriptors of grazoprevir as compared to simeprevir, its passive membrane permeability is significantly larger (5.9×10^6^ cm/s instead of 0.5×10^6^, Table S21). This indicates that the cyclization pattern of HCV protease inhibitors greatly influences the degree of passive membrane permeability, even if the degree of flexibility is seemingly unaffected by it. These findings contribute to the broader goal of rationally designing cell‐permeable drug candidates.

## 4 Materials and Methods

### 4.1 MicroED Structure Elucidation

The TEM grid was prepared following the previously published protocol [19, 36] by gentle agitation of commercial grazoprevir powders (Invivochem) mixed with a pre‐clipped 400‐mesh continuous‐carbon TEM grids (Ted Pella). Prior to mixing, the grid was glow‐discharged for 30 s on each side at 15 mA on the negative mode using PELCO easiGlow (Ted Pella). The grid was loaded into a Thermo–Fisher Talos Arctica TEM operating at 80 K and 200 kV. MicroED datasets were automatically collected using SerialEM following the previously published protocol [36]. Each dataset was continuously recorded on a Thermo–Fisher Falcon III detector at an electron dose rate of 0.01 e^−^/(Å^2^·s) and 0.5 s exposure per frame as the sample stage was continuously rotating from −68° to +70° at 2 degrees per second. MicroED datasets were processed following the previously published protocol [17, 18]. The datasets were initially processed using an in‐house developed Python script [36] for automatic image conversion, indexing, integration and scaling. The information on completeness and resolution produced by our automatic script offered a guideline for which datasets could be manually processed to improve the processing statistics. The datasets with over 80% completeness were manually reprocessed in XDS [37, 38] for refined indexing, integration and scaling. The reflection file was prepared from a single crystal dataset with an overall completeness of 92.3% using XPREP (Bruker), and the structure was solved by SHELXD [39] in the orthorhombic space group P2_1_2_1_2_1_ with the unit cell parameters of a = 6.85 Å, b = 17.45 Å, c = 34.51 Å, α = β = ɣ = 90°, followed by refinement in SHELXL [40, 41] using electron scattering factors. Unless specified, hydrogen atoms were located at the geometrically idealized positions and refined using riding model. MicroED data collection, data processing and structure refinement statistics are provided in the Supporting Information (Table S1).

### 4.2 NMR Ensemble Determination

Grazoprevir (Selleckchem) was studied at 3 mM in DMSO‐*d_6_* and CDCl_3_. All spectra were recorded on a 900 MHz Bruker Avance III HD NMR spectrometer equipped with a 5 mm TCI cryogenic probe at 25 °C. For the assignment of ^1^H and ^13^C resonances ^1^H, COSY, TOCSY, HSQC, HMBC, and NOESY spectra were recorded. NOE‐based interproton distances were derived from NOE build‐up rates by the initial rate approximation [42, 43]. For each solvent, a series of NOESY spectra with various mixing times between 100 and 700 ms, with 100 ms intervals, were recorded without solvent suppression, a relaxation delay of 2.5 s, 512 points in f1, 4096 points in f2, 16 transients and a spectral window of 12 ppm. Cross‐peak intensities were normalized according to the Peak Amplitude Normalization for Improved Cross‐relaxation (PANIC) method [43], prior to distance determination according to ((cross‐peak_ab_ × cross‐peak_ba_)/(diagonal‐peak_a_ × diagonal‐peak_b_))^1/2^. [42] The normalized intensities were plotted against the mixing time, from which the slope of the curve corresponds to the NOE build‐up rate (*σ*). The NOE build‐up rates were used for distance determination by *r*_ij_ = *r*_ref_ × (*σ*_ref_/*σ*_ij_)^1/6^, where *r*_ij_ and *r*_ref_ are the distance between H_a_ and H_b_ and the distance of a reference proton‐pair respectively, and *σ*_ij_ and *σ*_ref_ the corresponding NOE build‐up rates. The geminal proton‐pair at position 2 was used as a reference proton‐pair for the DMSO‐*d_6_* data and at position 13 for the CDCl_3_ data, both were referenced to 1.78 Å [44]. Other geminal proton‐pairs within the compound served a quality check for the reference proton‐pair. Only distances obtained from build‐up rates based on at least four NOESY spectra and with a coefficient of determination (*R* ^2^) ≥0.90 (typically ≥0.95) were used for ensemble determination. Scalar ^3^ *J*_HH_ couplings were extracted from the ^1^H spectrum. NMR signal assignment of grazoprevir, a list of interproton‐distances and coupling constant used for ensemble determination are provided in the Supporting Information (Tables S2–S4).

The solution ensembles of grazoprevir were elucidated by the NAMFIS algorithm [27]. Theoretical distances involving a methyl group were averaged according to d = ((d_a_^−6^+d_b_^−6^+d_c_^−6^)/3)^−1/6^, overlapping protons according to d = (d_a_^−6^+d_b_^−6^)^−1/6^, [45] and theoretical coupling constant were calculated according to the Karplus equation [46, 47]. The obtained solution was validated by excluding up to 10% of the experimental data‐points at the time from the deconvolution and by adding up to ±10% of experimental distances to the distances. The exclusion of data‐points was achieved by giving them an excessively large error, thereby making their contribution to the deconvolution calculation negligible. This process was repeated until all experimental data‐points were excluded at least once.

The theoretical input ensemble for the NAMFIS analysis was generated by Monte Carlo Multiple Minima (MCMM) conformational searches. In total eight conformational searched using four different force‐fields (OPLS‐2005, OPLS3, AMBER* and MMFF) with two different solvation models (CHCl_3_ and H_2_O) were run. Each search was performed using a maximum of 50,000 steps, an energy window of 42 kJ/mol, RMSD cut‐off of 2.0 Å and the Polak–Ribiere type conjugate gradient (PRCG) was selected with a maximum of 5,000 iterations. In addition to these conformation searches a macrocycle conformation sampling (MCS) was performed using OPLS‐2005 with GS/SA (water) as electrostatic treatment, making use of 5 000 large‐scale low‐mode (LLMOD) search steps, an energy window of 10 kcal/mol and an RMSD cut‐off of 2.0 Å. The resulting conformations were combined and redundant conformers were eliminated by comparison of all heavy atom positions, using an RMSD cut‐off of 2.0 Å. Crystal structures obtained from the PDB (IDs: 3SUD, 3SUE, 3SUF, 3SUG, 6C2M, and 6P6Q) [32], together with the MicroED structure were added to the combined and reduced calculated ensemble to generate the final input ensemble.

### 4.3 Molecular Descriptors and Single Point Energy Calculations

The SA 3D PSA for all conformations were calculated as previously described using PyMOL, [4, 6] and VEGA ZZ (version 3.2.3) [48, 49]. The *R*_gyr_ for all conformations were calculated in PyMOL (Supporting Information page 12). Partial charges and solvation energies were determined by B3LYP‐D3/6‐31** using the Single Point Energy (SPE) tool in the Jaguar [50] module as available in the Maestro suite (Schrödinger), after which the SA 3D PSA was calculated in PyMOL using a solvent probe radius of 1.4 Å and partial charges greater than 1 or smaller than −1 were included in the calculation [4]. The VEGA ZZ calculations were performed using comparable settings to the PyMOL calculations by using a probe radius set to 1.4 Å [49]. The Poisson–Boltzmann finite elements (PBF) water solvent model was used for the determination of the solvation energies for the MicroED, polar solution ensemble and target‐bound X‐ray crystal structures. For the apolar ensemble chloroform was used instead.

## Conflicts of Interest

The authors declare no conflicts of interest.

## Supporting information



Supporting Information containing additional experimental details, MicroED and NMR data collection and statistics, Figures S1–S13 and Tables S1–S21.
**Supporting File 1**: chem70554‐sup‐0001‐SuppMat.docx.

## Data Availability

We have deposited the original NMR spectroscopic data (FIDs) and the solution conformational ensemble (mol2) for this manuscript to the open access repository Zenodo with https://doi.org/10.5281/zenodo.17832871 The MicroED structure has been deposited in the Cambridge Structural Database under the CCDC deposition number 2468746 for grazoprevir.
