# Supplementary material for: Conformational Trajectory of the Molecular Chameleon Grazoprevir From Formulation to Target‐Bound
Source: Chemistry. 2025 Dec 18;32(8):e02256. doi: 10.1002/chem.202502256 (PMC12929930; doi:10.1002/chem.202502256)
Supplement: Supplementary file 1 — Supporting Information containing additional experimental details, MicroED and NMR data collection and statistics, Figures S1–S13 and Tables S1–S21. Supporting File 1: chem70554‐sup‐0001‐SuppMat.docx. [file CHEM-32-e02256-s001.pdf]

Conformational Trajectory of the Molecular Chameleon Grazoprevir from Formulation to Target-bound

Lianne H. E. Wieske,[1] Guanhong Bu,[2] Máté Erdélyi,[1] Jan Kihlberg,[1] Tamir Gonen[3,4] and Emma Rova Danelius[2,$]

1. Department of Chemistry – BMC and the Center of Excellence for the Chemical Mechanisms of Life, Uppsala University, Husargatan 3, 75237 Uppsala, Sweden.

2. Department of Chemistry, University of California Riverside, 501 Big Springs Rd, Riverside, CA, 92521, USA.

3. Department of Biological Chemistry & Physiology, University of California Los Angeles, 615 Charles E. Young Drive South, Los Angeles, CA 90095, USA.

4. Howard Hughes Medical Institute, University of California Los Angeles, 615 Charles E. Young Drive South, Los Angeles, CA 90095, USA.

$ Correspondence to E.R.D emmar@ucr.edu

Content

MicroED data S2
NMR data S4
Structure comparison S12
Cell permeability and solubility data S39
Literature data S40
Chameleonicity S41
References S42

MicroED data

Table S1. MicroED data collection, data processing and refinement statistics for grazoprevir.

Stoichiometric formula	$C_{38}H_{50}N_6O_9S$
Radiation wavelength (Å)	0.0251
Temperature (K)	80
Number of crystals	1
Crystal description	plate
Resolution (Å)	50.0 - 1.0
Crystal system	orthorhombic
Space group	$P2_12_12_1$
a (Å)	6.85
b (Å)	17.45
c (Å)	34.51
α (°)	90
β (°)	90
ɣ (°)	90
Z	4
Total reflections	10,780
Unique reflections	2,352
R_{obs} (%)	17.7
R_{meas} (%)	20.2
I/σI	6.47
$CC_{1/2}$ (%)	99.5
Completeness (%)	92.3
R_1	0.1585
wR_2	0.4153
GooF	1.288
CSD deposition ID	2468746

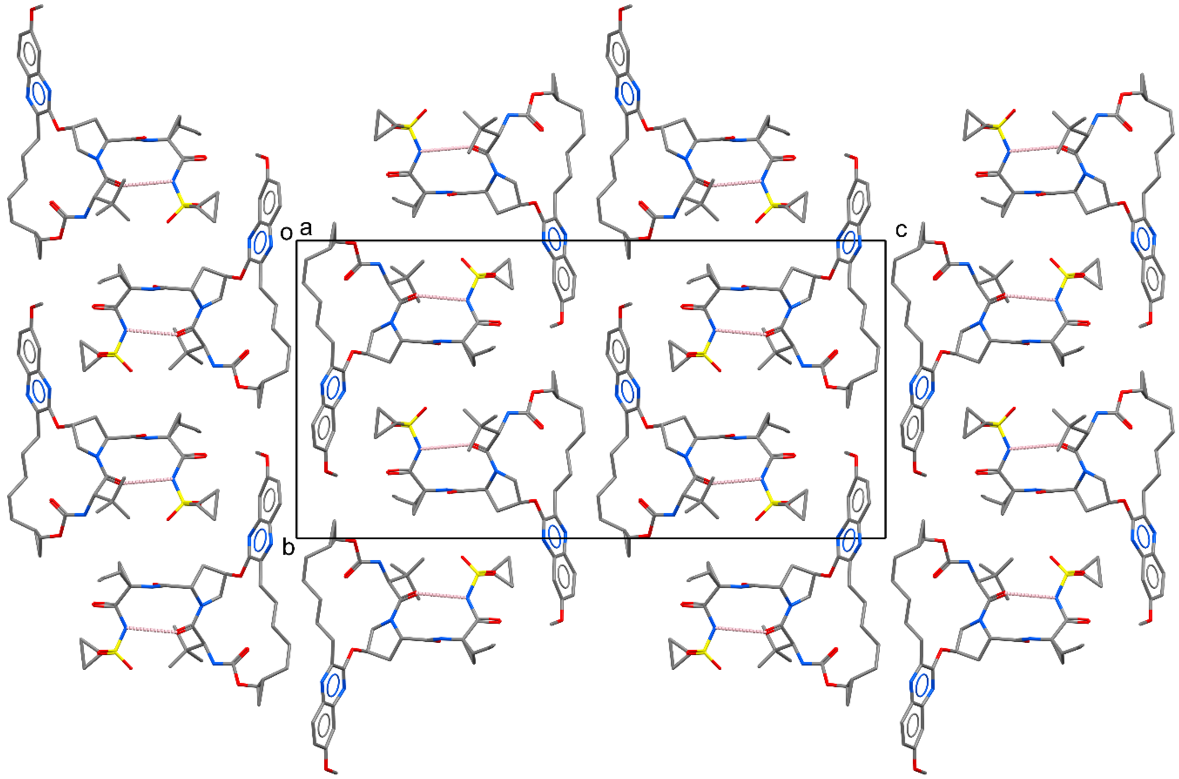

Figure S1. Molecular packing of grazoprevir in the unit cell as viewed along the crystallographic a-axis. Atom color: C, gray; N, blue; O, red; S, yellow. All hydrogen atoms are omitted for clarity. Intramolecular hydrogen bonds are shown in pink dashed lines.

NMR data

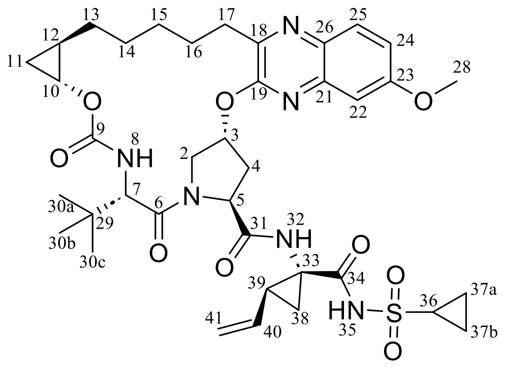

Figure S2. Grazoprevir

Table S2. NMR assignment (ppm) of grazoprevir in DMSO-d_6 and $CDCl_3$.

Macro-cycle	^1H DMSO-d_6 (ppm)	^1H $CDCl_3$ (ppm)	^{13}C DMSO-d_6 (ppm)	^{13}C $CDCl_3$ (ppm)	Side chain	^1H DMSO-d_6 (ppm)	^1H $CDCl_3$ (ppm)	^{13}C DMSO-d_6 (ppm)	^{13}C $CDCl_3$ (ppm)
2	4.37; 3.98	4.53; 4.05	53.9	54.5	21	-	-	140.2	n.a.
3	5.83	6.00	74.8	74.2	22	7.20	7.14	105.8	105.8
4	2.48; 2.12	2.58; 2.46	34.3	34.9	23	-	-	159.7	160.4
5	4.31	4.30	58.5	59.7	24	7.23	7.20	118.1	119.0
6	-	-	156.8	157.1	25	7.82	7.85	128.8	128.8
7	4.15	4.41	59.6	59.1	26	-	-	133.3	140.8
8-NH	7.15	5.32	-	-	28	3.90	3.94	55.5	55.5
9	-	-	170.5	172.2	29	-	-	33.9	34.9
10	3.61	3.76	54.3	55.1	30	1.01	1.08	26.2	26.4
11	0.85; 0.46	0.93; 0.48	10.6	11.1	31	-	-	172.2	n.a.
12	0.85	1.01	18.6	18.5	32-NH	8.80	6.63	-	-
13	1.70; 0.65	1.76; 0.68	37.2	30.6	33	-	-	40.7	41.6
14	1.48; 1.24	1.53	28.3	29.4	34	-	-	168.9	168.3
15	1.63; 1.48	1.70; 1.49	27.6	28.1	35-NH	10.45	10.05	-	-
16	1.82; 1.56	1.80; 1.63	27.2	28.3	36	2.93	2.91	30.5	30.9
17	2.88; 2.78	2.87; 2.80	32.5	33.9	37	1.08; 1.01	1.34; 1.03	5.5	6.2
18	-	-	148.0	150.0	38	1.67; 1.33	1.97; 1.49	22.0	23.1
19	-	-	154.5	n.a.	39	2.12	2.08	33.7	35.6
					40	5.59	5.79	133.5	132.5
					41	5.18; 5.07	5.21; 5.12	117.6	118.5

Atoms that could not be definitively assigned are indicated by n.a. (not assigned)

Table S3. Interproton distances (Å) for grazoprevir derived from NOE build-ups in DMSO-d_6.

No.	Proton i	Proton j	δ_i (ppm)	δ_j (ppm)	σ_{ij}	R2	Distance r_{ij} (Å)
1	3	5	5.83	4.31	0.0000105	0.99	3.53
2	22	3	7.20	5.83	0.0000087	0.99	3.64
3	3	7	5.83	4.15	0.0000043	0.99	4.10
4	40	38a	5.59	1.67	0.0000498	0.99	2.72
5	40	38b	5.59	1.33	0.0000944	0.99	2.45
6	5	4a	4.31	2.48	0.0002057	0.99	2.15
7	40	41b	5.59	5.07	0.0000517	0.99	2.70
8	3	4a	5.83	2.48	0.0001457	0.99	2.28
9	3	2a	5.83	4.37	0.0001013	0.99	2.42
10	3	2b	5.83	3.98	0.0001678	0.99	2.22
11	2a	7	4.37	4.15	0.0003374	0.99	1.98
12	7	2b	4.15	3.98	0.0001387	0.99	2.29
13	10	11b	3.61	0.46	0.0000925	0.99	2.46
14	10	13b	3.61	0.65	0.0000753	0.99	2.54
15	10	13a	3.61	1.70	0.0000149	0.99	3.33
16	10	15a	3.61	1.63	0.0000271	0.99	3.01
17	35-NH	5	10.45	4.31	0.0000061	0.95	3.87
18	32-NH	38b	8.80	1.33	0.0000385	0.99	2.84
19	32-NH	5	8.80	4.31	0.0002228	0.99	2.12
20	32-NH	2b	8.80	3.98	0.0000015	0.96	4.91
21	8-NH	10	7.15	3.61	0.0000098	0.98	3.57
22	8-NH	2a	7.15	4.37	0.0000048	0.98	4.02
23	8-NH	2b	7.15	3.98	0.0000021	0.98	4.61
24	2b	4a	3.98	2.48	0.0000368	0.98	2.86
25	17b	16b	2.78	1.56	0.0000280	0.97	3.00
26	13a	11b	1.70	0.46	0.0000431	0.99	2.79
27	13b	11b	0.65	0.46	0.0000968	0.98	2.44
28	15a	11b	1.63	0.46	0.0000039	0.97	4.16
29	13a	15a	1.70	1.63	0.0000679	0.97	2.58
30	15a	13b	1.63	0.65	0.0000926	0.99	2.45
31	22	28	7.20	3.90	0.0000552	0.99	2.68
32	24	28	7.23	3.90	0.0000066	0.99	3.81
33	7	10	4.15	3.61	0.0000013	0.96	5.01
34	5	2b	4.31	3.98	0.0000172	0.99	3.25
35	40	41a	5.59	5.18	0.0000218	0.96	3.12
36	22	4a	7.20	2.48	0.0000027	0.99	4.42
37	17a	16a	2.88	1.82	0.0000227	0.99	3.10
38	39	38a	2.12	1.67	0.0000323	0.97	2.93
39	39	38b	2.12	1.33	0.0000673	0.99	2.59
40	40	39	5.59	2.12	0.0000181	0.99	3.22
41	41a	39	5.18	2.12	0.0000458	0.99	2.76
42	41b	39	5.07	2.12	0.0000112	0.98	3.49

43	17a	14a	2.88	1.48	0.0000145	0.99	3.34
44	17b	14a	2.78	1.48	0.0000091	0.99	3.61
45	5	4b	4.31	2.12	0.0000433	0.98	2.79
46	10	11a	3.61	0.85	0.0000430	0.99	2.79
47	10	14a	3.61	1.48	0.0000051	0.99	3.97
48	2a	4b	4.37	2.12	0.0000130	0.98	3.40
49	13a	12	1.70	0.85	0.0000700	0.99	2.57
50	15a	12	1.63	0.85	0.0000144	0.98	3.35
51	14a	12	1.48	0.85	0.0000429	0.99	2.79
52	14a	13b	1.48	0.65	0.0001185	0.99	2.36
53	16a	15b	1.82	1.48	0.0000309	0.97	2.95
54	16b	14a	1.56	1.48	0.0000477	0.95	2.74
55	32-NH	39	8.80	2.12	0.0000736	0.99	2.55
56	3	4b	5.83	2.12	0.0000919	0.99	2.46
57	2b	4b	3.98	2.12	0.0000406	0.99	2.82
58	13a	14a	1.70	1.48	0.0000593	0.99	2.64
59	12	13b	0.85	0.65	0.0000614	0.98	2.63
Ref.	2a	2b	4.37	3.98	0.0006369	0.99	1.78
Ref.	4a	4b; 39	2.48	2.12	0.0005708	0.99	1.81
Ref.	11a; 12	11b	0.85	0.46	0.0003839	0.99	1.94
Ref.	13a	13b	1.70	0.65	0.0002566	0.99	2.07
Ref.	15a	14a; 15b	1.63	1.48	0.0001680	0.99	2.22
Ref.	16a	16b	1.82	1.56	0.0002487	0.99	2.08
Ref.	37a	30; 37b	1.08	1.01	0.0000652	0.99	2.60
Ref.	38a	38b	1.67	1.33	0.0000790	0.99	2.52
Ref.	41a	41b	5.18	5.07	0.0004240	0.99	1.90

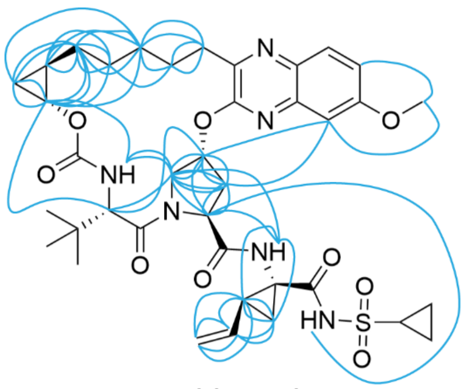

Figure S3. NMR-data coverage of grazoprevir in DMSO-d_6, NOE-based distances are shown in blue.

Table S4. Interproton distances (Å) derived from NOE build-ups and the scalar coupling for grazoprevir in $CDCl_3$ used for the NAMFIS analysis.

No.	Proton i	Proton j	δ_i (ppm)	δ_j (ppm)	σ_{ij}	R2	Distance r_{ij} (Å)
1	2a	3	4.53	6.00	0.00000065	0.99	3.53
2	2b	4b	4.05	2.46	0.00000173	0.94	3.00
3	3	4a	6.00	2.58	0.00000344	0.99	2.68
4	4a	5	2.58	4.30	0.00000666	0.94	2.40
5	5	32-NH	4.30	6.63	0.00000587	0.94	2.45
6	7	8-NH	4.41	5.32	0.00000059	0.92	3.59
7	10	13b	3.76	0.68	0.00000430	0.95	2.58
8	10	14ab	3.76	1.53	0.00000130	0.95	3.15
9	10	38b; 15b	3.76	1.49	0.00000056	0.90	3.62
10	10	40	3.76	5.79	0.00000018	0.99	4.38
11	12	14ab	1.01	1.53	0.00000175	0.97	3.00
12	13a	15b	1.76	1.49	0.00001117	0.96	2.20
13	13b	12	0.68	1.01	0.00000107	0.98	3.25
14	15a	17a	1.70	2.87	0.00000717	0.91	2.37
15	15a	17b	1.70	2.80	0.00000186	0.97	2.97
16	16a	17a	1.80	2.87	0.00000595	0.96	2.44
17	16a	17b	1.80	2.80	0.00000343	0.99	2.68
18	16b	17a	1.63	2.87	0.00000317	0.99	2.71
19	16b	17b	1.63	2.80	0.00001618	0.94	2.07
20	17a	15b	2.87	1.49	0.00000468	0.94	2.54
21	17b	15b	2.80	1.49	0.00000195	0.92	2.94
22	22	28	7.14	3.94	0.00000925	0.98	2.27
23	24	28	7.20	3.94	0.00000339	0.98	2.68
24	32-NH	38b	6.63	1.49	0.00000203	0.92	2.92
25	32-NH	39	6.63	2.08	0.00000329	0.94	2.70
26	35-NH	32-NH	10.05	6.63	0.00000082	0.92	3.40
27	35-NH	36	10.05	2.91	0.00000073	0.98	3.47
28	39	38b	2.08	1.49	0.00000895	0.94	2.28
29	39	41a	2.08	5.21	0.00000646	0.92	2.41
30	38a	40	1.98	5. 79	0.00000414	0.93	2.60
31	38a	41a	1.98	5.21	0.00000178	0.92	2.99
Ref.	13a	13b	1.76	0.68	0.00003981	0.99	1.78
Ref.	4a	4b	2.58	2.46	0.00001313	0.94	2.14
Ref.	11a	11b	0.93	0.48	0.00000354	0.94	2.66
Ref.	16a	16b	1.80	1.63	0.00002019	0.99	1.99
Ref.	38a	38b	1.97	1.49	0.00001528	0.99	2.09

Scalar coupling

Proton i	Proton j	δ_i (ppm)	δ_j (ppm)	$^3J_{HH}$ (Hz)
10	12	3.76	1.01	3.0

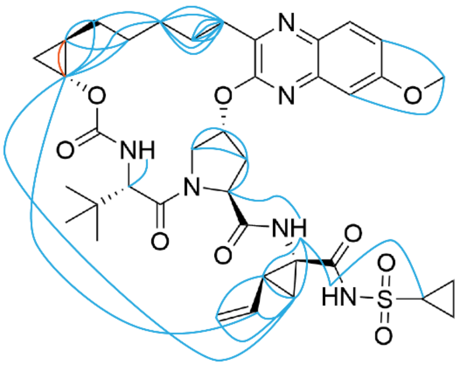

Figure S4. NMR-data coverage of grazoprevir in $CDCl_3$, NOE-based distances are shown in blue and $^3J_{HH}$ in orange.

Table S5. Macrocycle crystal structures from the Protein Data Bank (PDB) and the Cambridge Structural Database (CSD), added to the input ensembles and included in the NAMFIS analysis.

PDB	Organism	# of chains
3SUD	Hepatitis C virus 1a	4
3SUE	Hepatitis C virus 1a	4
3SUF	Hepatitis C virus 1a	4
3SUG	Hepatitis C virus 1a	1
6C2M	*Hepacivirus hominis*	4
6P6Q	Hepatitis C virus (isolate 1)	2

Table S6. Results of the MacroModel conformational searches.

		Number of conformations		
**Solvent**	**Force Field**	**Total[a]**	**Within 12.6 kJ/mol[b]**	**Final ensemble[c]**
**$CHCl_3$**	OPLS-2005	35	8	
	AMBER*	196	67	
	OPLS3	58	16	
	MMFF	105	42	
**H_2O**	OPLS-2005	38	6	84
	AMBER*	140	26	
	OPLS3	53	12	
	MMFF	148	78	
	MCS[d]	23		

[a]Total number of conformations found; [b]Number of conformations found within 12.6 kJ/mol from the energy minimum; [c]Final ensemble following a redundant conformer elimination with a RMSD cutoff of 2.0 Å for all heavy atoms including -OH and -NH and addition of the PDB and MicroED structures.

Table S7. Result of the NAMFIS-analyses for grazoprevir in DMSO-d_6 and $CDCl_3$

**DMSO-d_6**		**$CDCl_3$**	
**Conf. No.**	**Population (%)**	**Conf. No.**	**Population (%)**
**1**[a]	30	**1**[a]	4
**2**[b]	25	**9**[g]	25
**3**[c]	13	**10**	21
**4**[d]	10	**11**[h]	18
**5**	8	**12**	11
**6**[e]	7	**13**	9
**7**[f]	4	**14**	8
**8**	3	**15**	2
		**16**	2

X-ray structure, PDB codes: [a]6C2M chain M, [b]6C2M chain H, [c]3SUD chain E, [d]3SUD chain G, [e]3SUE chain K, [f]6P6Q chain J, [g]MicroED structure and [h]3SUG chain B.

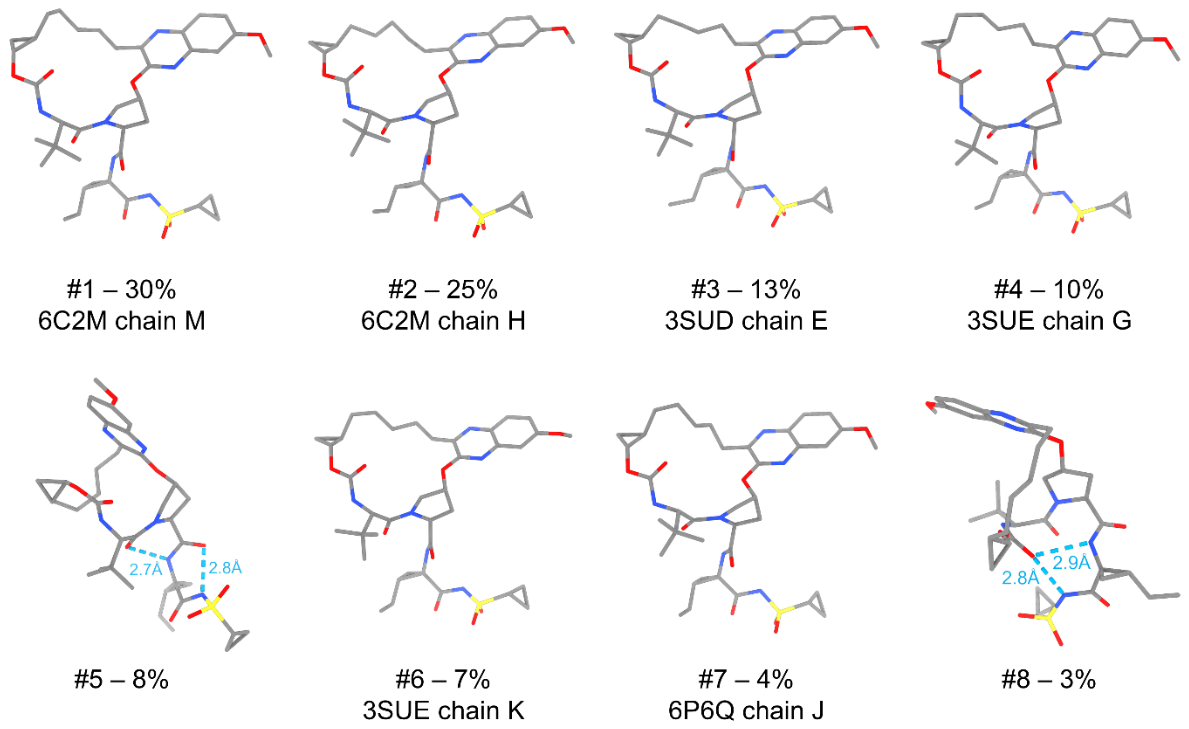

Figure S5. The solution conformations of grazoprevir in DMSO-d_6 as selected by the NAMFIS-analysis with their corresponding populations (Table S7), intramolecular hydrogen bonds are indicated by light blue dotted lines. Hydrogens are omitted for clarity.

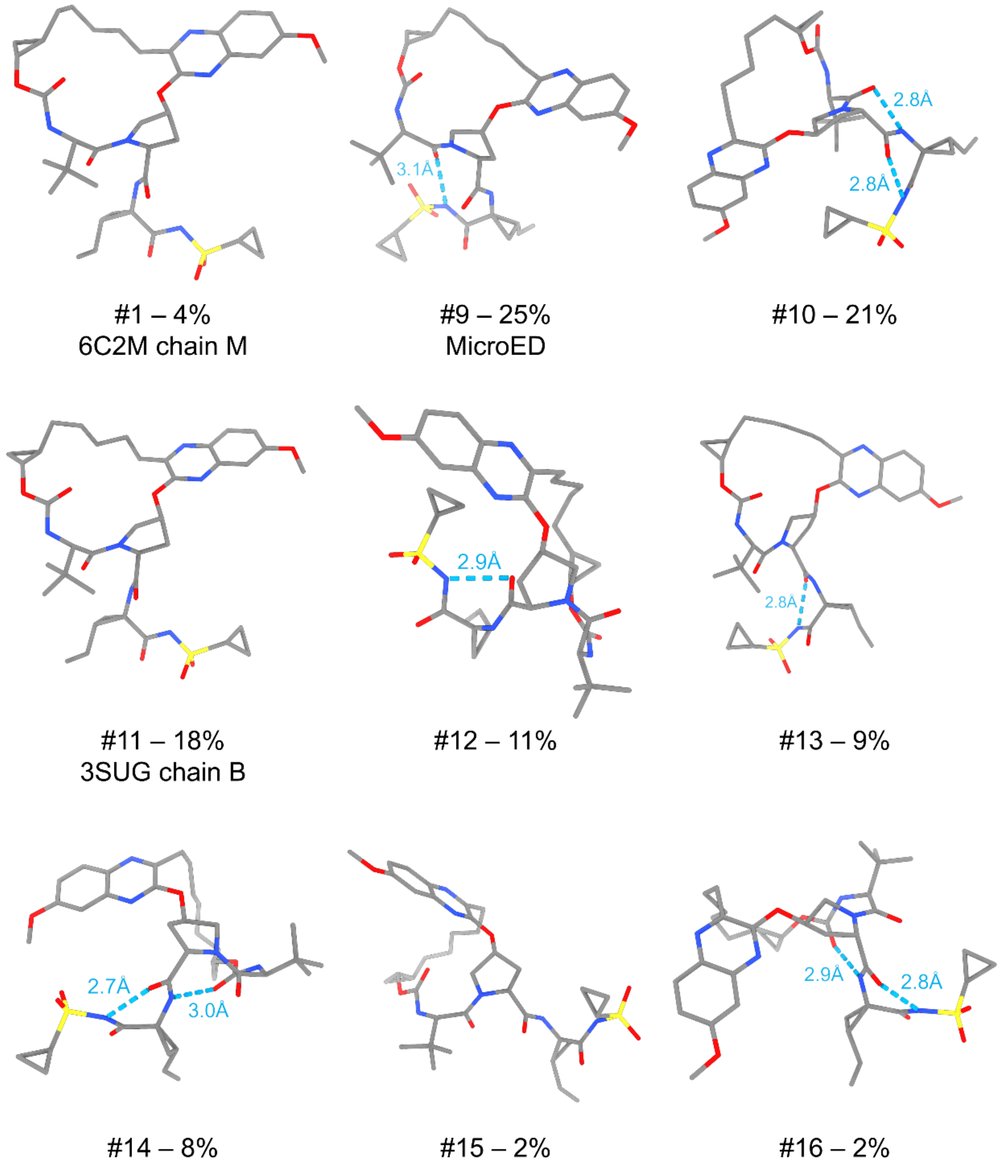

Figure S6. The solution conformations of grazoprevir in $CDCl_3$ as selected by the NAMFIS-analysis with their corresponding populations (Table S7), intramolecular hydrogen bonds are indicated by light blue dotted lines. Hydrogens are omitted for clarity.

Table S8. Experimentally determined and back-calculated (NAMFIS) interproton distances (Å) of grazoprevir in DMSO-d_6.

Exp.	Calc.	Exp.	Calc.	Exp.	Calc.	Exp.	Calc.
3.53	3.69	3.01	3.21	2.68	2.44	2.79	2.61
3.64	4.26	3.87	4.20	3.81	3.64	3.97	3.69
4.10	4.53	2.84	2.52	5.01	4.98	3.40	3.65
2.72	2.59	2.12	2.21	3.25	3.59	2.57	2.61
2.45	2.79	4.91	4.95	3.12	2.85	3.35	3.28
2.15	2.37	3.57	4.02	4.42	5.01	2.79	2.65
2.70	2.37	4.02	4.11	3.10	2.77	2.36	2.49
2.28	2.52	4.61	4.70	2.93	2.54	2.95	2.62
2.42	2.49	2.86	3.34	2.59	2.45	2.74	2.46
2.22	2.41	3.00	2.78	3.22	2.97	2.55	2.87
1.98	2.09	2.79	2.68	2.76	2.52	2.46	2.40
2.29	2.47	2.44	2.58	3.49	3.53	2.82	2.78
2.46	2.35	4.16	4.57	3.34	3.47	2.64	2.57
2.54	2.56	2.58	2.65	3.61	3.44	2.63	2.70
3.33	3.18	2.45	2.64	2.79	2.57		

Table S9. Experimentally determined and back-calculated (NAMFIS) interproton distances (Å) and scalar coupling of grazoprevir in $CDCl_3$.

Exp.	Calc.	Exp.	Calc.	Exp.	Calc.	Exp. $^3J_{HH}$	Calc. $^3J_{HH}$
3.53	2.73	2.44	2.57	2.99	3.07	3.0	3.2
3.00	3.07	2.68	2.73				
2.68	2.73	2.71	2.73				
2.40	2.31	2.07	2.37				
2.45	2.38	2.54	2.71				
3.59	2.84	2.94	2.81				
2.58	2.60	2.27	2.49				
3.15	2.97	2.68	2.88				
3.62	3.80	2.92	2.65				
4.38	4.70	2.70	2.69				
3.00	2.91	3.40	3.23				
2.20	2.60	3.47	3.37				
3.25	2.93	2.28	2.38				
2.37	2.44	2.41	2.60				
2.97	3.03	2.60	2.54				

Structure comparison

The radius of gyration for the various conformations were calculated using the script written by Tsjerk Wassenaar as posted on wiki.pymol.org.

```
from pymol import cmd
import math

def rgyrate(selection='(all)', quiet=1):
    '''
DESCRIPTION

    radius of gyration

USAGE

    rgyrate [ selection ]
    '''
    try:
        from itertools import izip
    except ImportError:
        izip = zip
    quiet = int(quiet)
    model = cmd.get_model(selection).atom
    x = [i.coord for i in model]
    mass = [i.get_mass() for i in model]
    xm = [(m*i,m*j,m*k) for (i,j,k),m in izip(x,mass)]
    tmass = sum(mass)
    rr = sum(mi*i+mj*j+mk*k for (i,j,k),(mi,mj,mk) in izip(x,xm))
    mm = sum((sum(i)/tmass)**2 for i in izip(*xm))
    rg = math.sqrt(rr/tmass - mm)
    if not quiet:
        print("Radius of gyration: %.2f" % (rg))
    return rg

cmd.extend("rgyrate", rgyrate)
```

Table S10. The solvent accessible 3D polar surface area (SA 3D PSA) and radius of gyration (R_{gyr}) of the grazoprevir conformations found by different methods as well as the solvation energies as determined by the single point energy tool in Jaguar. The SA 3D PSA was calculated in PyMOL according to Rossi Sebatiano *et al*[1–3] and VEGA ZZ[4,5].

Target bound	SA 3D PSA (Å^2) PyMOL	SA 3D PSA (Å^2) VEGA ZZ	R_{gyr} (Å)	Energy (kcal/mol)
**3SUD Chain E**	176	177	5.60	-41.0
**3SUD Chain H**	172	173	5.61	-46.3
**3SUD Chain J**	173	171	5.60	-39.4
**3SUD Chain L**	169	169	5.64	-37.9
**3SUE Chain E**	173	175	5.57	-39.6
**3SUE Chain G**	165	169	5.55	-39.9
**3SUE Chain I**	179	176	5.64	-38.6
**3SUE Chain K**	174	173	5.66	-42.1
**3SUF Chain E**	169	168	5.54	-39.0
**3SUF Chain G**	185	191	5.61	-44.2
**3SUF Chain J**	179	179	5.57	-39.9
**3SUF Chain L**	165	170	5.57	-39.9
**3SUG Chain B**	172	170	5.61	-42.8
**6C2M Chain F**	187	187	5.72	-36.8
**6C2M Chain H**	197	199	5.72	-37.5
**6C2M Chain K**	192	193	5.71	-37.1
**6C2M Chain M**	198	198	5.75	-37.9
**6P6Q Chain C**	205	200	5.85	-39.9
**6P6Q Chain J**	196	195	5.78	-39.1
**Average**	180	181	5.65	-39.9

MicroED from Vendor	SA 3D PSA (Å^2) PyMOL	SA 3D PSA (Å^2) VEGA ZZ	R_{gyr} (Å)	Energy (kcal/mol)
**MicroED**	174	176	5.57	-31.3

Polar NMR ensemble (population)	SA 3D PSA (Å^2) PyMOL	SA 3D PSA (Å^2) VEGA ZZ	R_{gyr} (Å)	Energy (kcal/mol)
**1 - 6C2M M (30%)**	198	198	5.75	-37.9
**2 - 6C2M H (25%)**	197	199	5.72	-37.5
**3 - 3SUD E (13%)**	176	177	5.60	-41.0
**4 - 3SUE G (10%)**	165	169	5.55	-39.9
**5 (8%)**	144	144	5.25	-25.2
**6 - 3SUE K (7%)**	174	173	5.66	-42.1
**7 - 6P6Q J (4%)**	196	195	5.78	-39.1
**8 (3%)**	172	164	5.02	-32.1
**Population average**	185	185	5.64	-37.5

Apolar NMR ensemble (population)	SA 3D PSA (Å^2) PyMOL	SA 3D PSA (Å^2) VEGA ZZ	R_{gyr} (Å)	Energy (kcal/mol)
**1 - 6C2M M (4%)**	198	198	5.75	-27.4
**9 - MicroED (25%)**	174	176	5.57	-23.8
**10 (21%)**	195	199	5.01	-22.3
**11 - 3SUG B (18%)**	172	170	5.61	-28.4
**12 (11%)**	210	210	4.92	-26.6
**13 (9%)**	179	181	5.68	-22.2
**14 (8%)**	131	132	5.07	-18.7
**15 (2%)**	173	170	5.52	-22.8
**16 (2%)**	163	167	5.27	-21.4
**Population average**	180	181	5.36	-24.1

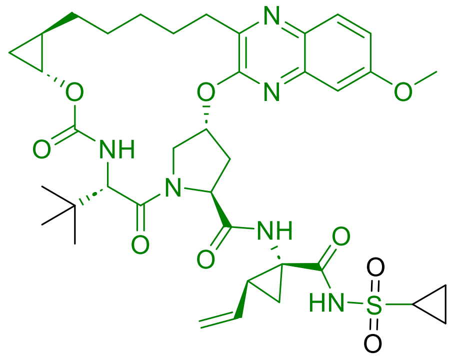

Figure S7. Structure of grazoprevir with atoms used for RMSD comparison highlighted in green.

Table S11. RMSD value for all non-hydrogen atoms and NH hydrogens (Figure S7) of the grazoprevir conformations found by MicroED, NMR, and target-bound X-ray crystallography. RMSD values of 0.500 Å or below are highlighted in green.

	**MicroED**	**DMSO -*d*$_6$ 1**	**DMSO -*d*$_6$ 2**	**DMSO -*d*$_6$ 3**	**DMSO -*d*$_6$ 4**	**DMSO -*d*$_6$ 5**	**DMSO -*d*$_6$ 6**	**DMSO -*d*$_6$ 7**	**DMSO -*d*$_6$ 8**	**CDCl3 1**	**CDCl3 9**	**CDCl3 10**	**CDCl3 11**	**CDCl3 12**	**CDCl3 13**	**CDCl3 14**	**CDCl3 15**	**CDCl3 16**
**MicroED**	0.000																	
**DMSO-*d*$_6$ 1**	2.158	0.000																
**DMSO-*d*$_6$ 2**	2.143	0.432	0.000															
**DMSO-*d*$_6$ 3**	2.094	0.510	0.375	0.000														
**DMSO-*d*$_6$ 4**	2.117	0.492	0.420	0.319	0.000													
**DMSO-*d*$_6$ 5**	3.129	2.927	2.944	2.880	2.818	0.000												
**DMSO-*d*$_6$ 6**	2.131	0.399	0.431	0.413	0.381	2.905	0.000											
**DMSO-*d*$_6$ 7**	2.175	0.461	0.427	0.464	0.456	2.958	0.438	0.000										
**DMSO-*d*$_6$ 8**	4.131	3.888	3.848	3.873	3.775	3.665	3.864	3.898	0.000									
**CDCl3 1**	2.158	**0.000**	0.432	0.510	0.492	2.927	0.399	0.461	3.888	0.000								
**CDCl3 9**	**0.000**	2.158	2.143	2.094	2.117	3.129	2.131	2.175	4.131	2.158	0.000							
**CDCl3 10**	2.875	2.071	2.147	2.111	2.047	2.689	2.009	2.127	4.066	2.071	2.875	0.000						

**CDCl3 11**	2.119	0.427	0.446	0.414	0.395	2.914	0.313	0.388	3.820	0.427	2.119	2.042	0.000					
**CDCl3 12**	3.961	3.421	3.451	3.428	3.394	4.272	3.327	3.517	3.932	3.421	3.961	3.313	3.327	0.000				
**CDCl3 13**	2.062	2.554	2.607	2.616	2.539	3.675	2.474	2.607	4.050	2.554	2.062	2.674	2.535	3.397	0.000			
**CDCl3 14**	3.857	3.078	3.185	3.159	3.040	3.585	3.014	3.199	4.002	3.078	3.857	2.740	3.036	2.088	2.858	0.000		
**CDCl3 15**	2.901	2.179	2.155	2.120	2.095	1.605	2.164	2.155	3.624	2.179	2.901	2.232	2.168	4.354	3.428	3.766	0.000	
**CDCl3 16**	3.261	3.313	3.403	3.405	3.293	3.644	3.246	3.420	3.551	3.313	3.261	3.136	3.296	2.746	1.956	1.862	3.794	0.000
**3SUD E**	2.094	0.510	0.375	**0.000**	0.319	2.880	0.413	0.464	3.873	0.510	2.094	2.111	0.414	3.428	2.616	3.159	2.120	3.405
**3SUD H**	2.157	0.427	0.407	0.417	0.340	2.867	0.369	0.360	3.782	0.427	2.157	2.116	0.326	3.466	2.572	3.111	2.080	3.334
**3SUD J**	2.139	0.453	0.381	0.317	0.363	2.866	0.291	0.484	3.796	0.453	2.139	2.078	0.395	3.336	2.543	3.062	2.110	3.287
**3SUD L**	2.110	0.413	0.406	0.426	0.382	2.874	0.220	0.459	3.847	0.413	2.110	2.028	0.336	3.343	2.469	3.012	2.147	3.242
**3SUE E**	2.127	0.432	0.365	0.280	0.277	2.810	0.314	0.403	3.782	0.432	2.127	2.048	0.356	3.390	2.552	3.066	2.053	3.291
**3SUE G**	2.117	0.492	0.420	0.319	**0.000**	2.818	0.381	0.456	3.775	0.492	2.117	2.047	0.395	3.394	2.539	3.040	2.095	3.293
**3SUE I**	2.173	0.444	0.479	0.414	0.418	2.886	0.215	0.413	3.848	0.444	2.173	2.016	0.306	3.373	2.518	3.039	2.113	3.270
**3SUE K**	2.131	0.399	0.431	0.413	0.381	2.905	**0.000**	0.438	3.864	0.399	2.131	2.009	0.313	3.327	2.474	3.014	2.164	3.246
**3SUF E**	2.061	0.503	0.456	0.240	0.310	2.845	0.416	0.511	3.858	0.503	2.061	2.063	0.429	3.390	2.550	3.071	2.123	3.319
**3SUF G**	2.148	0.399	0.368	0.268	0.358	2.835	0.391	0.415	3.826	0.399	2.148	2.070	0.400	3.452	2.627	3.147	2.029	3.380
**3SUF J**	2.152	0.504	0.482	0.408	0.358	2.955	0.385	0.436	3.844	0.504	2.152	2.098	0.433	3.345	2.519	3.062	2.217	3.303
**3SUF L**	2.156	0.409	0.481	0.458	0.371	2.853	0.255	0.499	3.819	0.409	2.156	1.992	0.406	3.311	2.469	2.937	2.138	3.181
**3SUG B**	2.119	0.427	0.446	0.414	0.395	2.914	0.313	0.388	3.820	0.427	2.119	2.042	**0.000**	3.327	2.535	3.036	2.168	3.296
**6C2M F**	2.043	0.499	0.431	0.413	0.462	2.931	0.449	0.431	3.910	0.499	2.043	2.163	0.460	3.515	2.586	3.223	2.181	3.421
**6C2M H**	2.143	0.432	**0.000**	0.375	0.420	2.944	0.431	0.427	3.848	0.432	2.143	2.147	0.446	3.451	2.607	3.185	2.155	3.403

**6C2M K**	2.165	0.437	0.373	0.441	0.420	3.024	0.356	0.307	3.813	0.437	2.165	2.148	0.345	3.398	2.569	3.121	2.276	3.359
**6C2M M**	2.158	**0.000**	0.432	0.510	0.492	2.927	0.399	0.461	3.888	**0.000**	2.158	2.071	0.427	3.421	2.554	3.078	2.179	3.313
**6P6Q C**	2.206	0.459	0.446	0.472	0.471	3.025	0.398	0.207	3.960	0.459	2.206	2.113	0.396	3.445	2.574	3.145	2.235	3.397
**6P6Q J**	2.175	0.461	0.427	0.464	0.456	2.958	0.438	**0.000**	3.898	0.461	2.175	2.127	0.388	3.517	2.607	3.199	2.155	3.420

Table S12. RMSD value for all non-hydrogen atoms and NH hydrogens (Figure S7) of the grazoprevir conformations found by target-bound X-ray crystallography. RMSD values of 0.500 Å or below are highlighted in green.

	**3SUD E**	**3SUD H**	**3SUD J**	**3SUD L**	**3SUE E**	**3SUE G**	**3SUE I**	**3SUE K**	**3SUF E**	**3SUF G**	**3SUF J**	**3SUF L**	**3SUG B**	**6C2M F**	**6C2M H**	**6C2M K**	**6C2M M**	**6P6Q C**	**6P6Q J**
**3SUD E**	0.000																		
**3SUD H**	0.417	0.000																	
**3SUD J**	0.317	0.411	0.000																
**3SUD L**	0.426	0.310	0.358	0.000															
**3SUE E**	0.280	0.301	0.263	0.327	0.000														
**3SUE G**	0.319	0.340	0.363	0.382	0.277	0.000													
**3SUE I**	0.414	0.365	0.286	0.302	0.325	0.418	0.000												
**3SUE K**	0.413	0.369	0.291	0.220	0.314	0.381	0.215	0.000											
**3SUF E**	0.240	0.426	0.366	0.401	0.308	0.310	0.423	0.416	0.000										
**3SUF G**	0.268	0.335	0.307	0.397	0.214	0.358	0.388	0.391	0.341	0.000									
**3SUF J**	0.408	0.441	0.336	0.434	0.354	0.358	0.383	0.385	0.442	0.436	0.000								
**3SUF L**	0.458	0.399	0.290	0.334	0.307	0.371	0.287	0.255	0.424	0.408	0.351	0.000							
**3SUG B**	0.414	0.326	0.395	0.336	0.356	0.395	0.306	0.313	0.429	0.400	0.433	0.406	0.000						
**6C2M F**	0.413	0.438	0.492	0.448	0.441	0.462	0.486	0.449	0.367	0.448	0.552	0.535	0.460	0.000					

**6C2MH**	0.375	0.407	0.381	0.406	0.365	0.420	0.479	0.431	0.456	0.368	0.482	0.481	0.446	0.431	0.000				
**6C2MK**	0.441	0.397	0.403	0.413	0.404	0.420	0.373	0.356	0.478	0.441	0.422	0.419	0.345	0.407	0.373	0.000			
**6C2MM**	0.510	0.427	0.453	0.413	0.432	0.492	0.444	0.399	0.503	0.399	0.504	0.409	0.427	0.499	0.432	0.437	0.000		
**6P6QC**	0.472	0.426	0.480	0.439	0.443	0.471	0.413	0.398	0.514	0.455	0.458	0.510	0.396	0.461	0.446	0.300	0.459	0.000	
**6P6QJ**	0.464	0.360	0.484	0.458	0.403	0.456	0.413	0.438	0.511	0.415	0.436	0.499	0.388	0.431	0.427	0.307	0.461	0.207	0.000

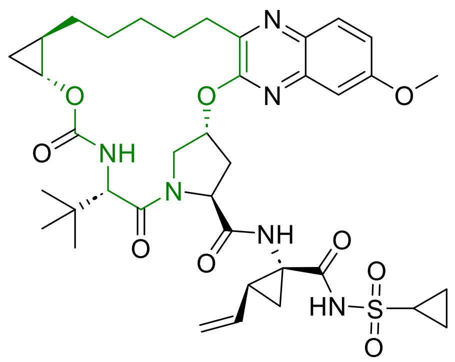

Figure S8. Structure of grazoprevir with atoms used for RMSD comparison highlighted in green.

Table S13. RMSD value for all non-hydrogen atoms of the macrocyclic core (Figure S8) of the grazoprevir conformations found by MicroED, NMR, and target-bound X-ray crystallography. RMSD values of 0.300 Å or below are highlighted in green.

	MicroED	DMSO-d_6 1	DMSO-d_6 2	DMSO-d_6 3	DMSO-d_6 4	DMSO-d_6 5	DMSO-d_6 6	DMSO-d_6 7	DMSO-d_6 8	CDCl3 1	CDCl3 9	CDCl3 10	CDCl3 11	CDCl3 12	CDCl3 13	CDCl3 14	CDCl3 15	CDCl3 16
**MicroED**	0.000																	
**DMSO-d_6 1**	0.494	0.000																
**DMSO-d_6 2**	0.505	0.588	0.000															
**DMSO-d_6 3**	0.439	0.577	0.387	0.000														
**DMSO-d_6 4**	0.434	0.563	0.390	0.294	0.000													
**DMSO-d_6 5**	1.517	1.537	1.485	1.366	1.343	0.000												
**DMSO-d_6 6**	0.153	0.500	0.510	0.385	0.373	1.444	0.000											
**DMSO-d_6 7**	0.495	0.649	0.589	0.539	0.504	1.658	0.488	0.000										
**DMSO-d_6 8**	1.136	1.176	1.244	1.200	1.088	1.835	1.096	1.195	0.000									
**CDCl3 1**	0.494	**0.000**	0.588	0.577	0.563	1.537	0.500	0.649	1.176	0.000								
**CDCl3 9**	**0.000**	0.494	0.505	0.439	0.434	1.517	0.153	0.495	1.136	0.494	0.000							
**CDCl3 10**	1.201	1.325	1.336	1.253	1.188	1.570	1.151	1.362	1.467	1.325	1.201	0.000						

**CDCl3 11**	0.273	0.551	0.587	0.468	0.478	1.588	0.260	0.428	1.087	0.551	0.273	1.191	0.000					
**CDCl3 12**	1.053	0.996	1.202	1.128	1.152	1.682	1.033	1.273	1.309	0.996	1.053	1.017	1.064	0.000				
**CDCl3 13**	0.549	0.768	0.808	0.779	0.706	1.679	0.541	0.800	0.999	0.769	0.549	0.956	0.539	1.006	0.000			
**CDCl3 14**	1.073	1.049	1.218	1.124	1.064	1.543	1.033	1.197	0.836	1.049	1.073	1.355	1.059	1.105	0.954	0.000		
**CDCl3 15**	0.923	0.964	0.845	0.826	0.780	0.902	0.887	1.061	1.415	0.964	0.923	1.187	1.020	1.265	1.075	1.136	0.000	
**CDCl3 16**	1.061	1.039	1.208	1.115	1.055	1.555	1.022	1.184	0.820	1.039	1.061	1.357	1.047	1.099	0.943	0.031	1.141	0.000
**3SUD E**	0.439	0.577	0.387	**0.000**	0.294	1.366	0.385	0.539	1.200	0.577	0.439	1.253	0.468	1.128	0.779	1.124	0.826	1.115
**3SUD H**	0.311	0.466	0.492	0.491	0.423	1.506	0.330	0.426	1.141	0.466	0.311	1.305	0.353	1.175	0.681	1.118	0.912	1.107
**3SUD J**	0.389	0.507	0.386	0.145	0.254	1.360	0.325	0.540	1.152	0.507	0.389	1.248	0.438	1.101	0.738	1.082	0.824	1.073
**3SUD L**	0.256	0.519	0.440	0.431	0.413	1.448	0.243	0.526	1.123	0.519	0.256	1.223	0.349	1.106	0.599	1.076	0.873	1.066
**3SUE E**	0.372	0.470	0.420	0.196	0.272	1.417	0.335	0.456	1.128	0.470	0.372	1.285	0.407	1.122	0.739	1.065	0.849	1.054
**3SUE G**	0.434	0.563	0.390	0.294	**0.000**	1.343	0.373	0.504	1.088	0.563	0.434	1.188	0.478	1.152	0.706	1.064	0.781	1.055
**3SUE I**	0.217	0.517	0.524	0.368	0.367	1.486	0.148	0.397	1.083	0.517	0.217	1.191	0.181	1.077	0.570	1.029	0.930	1.017
**3SUE K**	0.153	0.500	0.510	0.385	0.373	1.444	**0.000**	0.488	1.096	0.500	0.153	1.151	0.260	1.032	0.541	1.033	0.887	1.022
**3SUF E**	0.369	0.485	0.393	0.180	0.231	1.401	0.319	0.477	1.112	0.485	0.369	1.261	0.422	1.114	0.728	1.048	0.833	1.038
**3SUF G**	0.400	0.403	0.445	0.235	0.331	1.390	0.356	0.506	1.183	0.403	0.400	1.283	0.453	1.082	0.777	1.074	0.826	1.065
**3SUF J**	0.435	0.492	0.431	0.306	0.287	1.468	0.395	0.410	1.098	0.492	0.435	1.241	0.439	1.099	0.729	1.060	0.868	1.049
**3SUF L**	0.316	0.371	0.492	0.358	0.280	1.410	0.273	0.493	1.028	0.371	0.316	1.200	0.378	1.044	0.623	0.949	0.833	0.938
**3SUG B**	0.273	0.551	0.587	0.468	0.478	1.588	0.260	0.428	1.087	0.551	0.273	1.191	**0.000**	1.064	0.539	1.059	1.020	1.047
**6C2M F**	0.311	0.551	0.463	0.445	0.373	1.506	0.320	0.371	1.155	0.551	0.311	1.334	0.386	1.228	0.717	1.137	0.923	1.125
**6C2M H**	0.505	0.588	**0.000**	0.387	0.390	1.485	0.510	0.589	1.244	0.588	0.505	1.336	0.587	1.202	0.808	1.218	0.845	1.208

**6C2M K**	0.387	0.623	0.492	0.449	0.372	1.586	0.361	0.255	1.146	0.623	0.387	1.295	0.374	1.233	0.709	1.183	1.011	1.170
**6C2M M**	0.494	**0.000**	0.588	0.577	0.563	1.537	0.500	0.649	1.176	0.000	0.494	1.325	0.551	0.996	0.769	1.049	0.964	1.039
**6P6Q C**	0.458	0.647	0.562	0.478	0.481	1.624	0.423	0.188	1.222	0.647	0.458	1.301	0.399	1.223	0.763	1.223	1.055	1.210
**6P6Q J**	0.495	0.649	0.589	0.539	0.504	1.658	0.488	**0.000**	1.195	0.649	0.495	1.362	0.428	1.273	0.800	1.197	1.061	1.184

Table S14. RMSD value for all non-hydrogen atoms of the macrocyclic core (Figure S8) of the grazoprevir conformations found by target-bound X-ray crystallography. RMSD values of 0.300 Å or below are highlighted in green.

	**3SUD E**	**3SUD H**	**3SUD J**	**3SUD L**	**3SUE E**	**3SUE G**	**3SUE I**	**3SUE K**	**3SUF E**	**3SUF G**	**3SUF J**	**3SUF L**	**3SUG B**	**6C2M F**	**6C2M H**	**6C2M K**	**6C2M M**	**6P6Q C**	**6P6Q J**
**3SUD E**	0.000																		
**3SUD H**	0.491	0.000																	
**3SUD J**	0.145	0.451	0.000																
**3SUD L**	0.431	0.254	0.406	0.000															
**3SUE E**	0.196	0.378	0.182	0.389	0.000														
**3SUE G**	0.294	0.423	0.254	0.413	0.272	0.000													
**3SUE I**	0.368	0.323	0.322	0.295	0.305	0.367	0.000												
**3SUE K**	0.385	0.330	0.325	0.243	0.335	0.373	0.148	0.000											
**3SUF E**	0.180	0.400	0.149	0.376	0.099	0.231	0.305	0.319	0.000										
**3SUF G**	0.235	0.404	0.197	0.418	0.145	0.331	0.351	0.356	0.166	0.000									
**3SUF J**	0.306	0.400	0.323	0.410	0.236	0.287	0.359	0.395	0.239	0.291	0.000								
**3SUF L**	0.358	0.329	0.283	0.355	0.226	0.280	0.278	0.273	0.224	0.241	0.284	0.000							
**3SUG B**	0.468	0.353	0.438	0.349	0.407	0.478	0.181	0.260	0.422	0.453	0.439	0.378	0.000						
**6C2M F**	0.445	0.300	0.384	0.376	0.364	0.373	0.295	0.320	0.364	0.399	0.438	0.356	0.386	0.000					

**6C2M H**	0.387	0.492	0.386	0.440	0.420	0.390	0.524	0.510	0.393	0.445	0.431	0.492	0.587	0.463	0.000				
**6C2M K**	0.449	0.394	0.420	0.433	0.397	0.372	0.301	0.361	0.389	0.453	0.395	0.415	0.374	0.232	0.492	0.000			
**6C2M M**	0.577	0.466	0.507	0.519	0.470	0.563	0.517	0.500	0.485	0.403	0.492	0.371	0.551	0.551	0.588	0.623	0.000		
**6P6Q C**	0.478	0.455	0.485	0.484	0.441	0.481	0.350	0.423	0.446	0.476	0.409	0.490	0.399	0.380	0.562	0.213	0.647	0.000	
**6P6Q J**	0.539	0.426	0.540	0.526	0.456	0.504	0.397	0.488	0.477	0.506	0.410	0.493	0.428	0.371	0.589	0.255	0.649	0.188	0.000

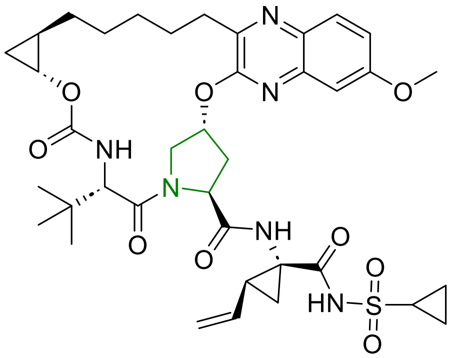

Figure S9. Structure of grazoprevir with atoms used for RMSD comparison highlighted in green.

Table S15. RMSD value for all non-hydrogen atoms of the proline ring (Figure S9) of grazoprevir conformations found by MicroED, NMR, and target-bound X-ray crystallography. RMSD values of 0.120 Å or below are highlighted in green.

	**MicroED**	**DMSO -d_6 1**	**DMSO -d_6 2**	**DMSO -d_6 3**	**DMSO -d_6 4**	**DMSO -d_6 5**	**DMSO -d_6 6**	**DMSO -d_6 7**	**DMSO -d_6 8**	**CDCl3 1**	**CDCl3 9**	**CDCl3 10**	**CDCl3 11**	**CDCl3 12**	**CDCl3 13**	**CDCl3 14**	**CDCl3 15**	**CDCl3 16**
**MicroED**	0.000																	
**DMSO-d_6 1**	0.082	0.000																
**DMSO-d_6 2**	0.088	0.045	0.000															
**DMSO-d_6 3**	0.093	0.082	0.041	0.000														
**DMSO-d_6 4**	0.073	0.070	0.043	0.044	0.000													
**DMSO-d_6 5**	0.090	0.072	0.031	0.017	0.042	0.000												
**DMSO-d_6 6**	0.052	0.081	0.067	0.063	0.051	0.060	0.000											
**DMSO-d_6 7**	0.114	0.057	0.038	0.069	0.070	0.062	0.102	0.000										
**DMSO-d_6 8**	0.311	0.378	0.372	0.357	0.340	0.359	0.314	0.405	0.000									
**CDCl3 1**	0.082	**0.000**	0.045	0.082	0.070	0.072	0.081	0.057	0.378	0.000								
**CDCl3 9**	**0.000**	0.082	0.088	0.093	0.073	0.090	0.052	0.114	0.311	0.082	0.000							
**CDCl3 10**	0.340	0.394	0.375	0.355	0.343	0.357	0.323	0.406	0.138	0.394	0.340	0.000						

**CDCl3 11**	0.070	0.062	0.057	0.063	0.062	0.055	0.059	0.088	0.340	0.062	0.070	0.355	0.000					
**CDCl3 12**	0.326	0.380	0.360	0.339	0.327	0.341	0.309	0.390	0.143	0.380	0.326	0.023	0.341	0.000				
**CDCl3 13**	0.314	0.376	0.363	0.345	0.330	0.346	0.308	0.395	0.070	0.376	0.314	0.069	0.336	0.075	0.000			
**CDCl3 14**	0.357	0.422	0.413	0.397	0.380	0.399	0.356	0.444	0.060	0.422	0.357	0.116	0.385	0.129	0.066	0.000		
**CDCl3 15**	0.054	0.087	0.084	0.081	0.073	0.077	0.043	0.120	0.305	0.087	0.054	0.325	0.049	0.311	0.304	0.351	0.000	
**CDCl3 16**	0.356	0.420	0.411	0.395	0.378	0.396	0.354	0.442	0.063	0.420	0.356	0.109	0.383	0.122	0.060	0.008	0.350	0.000
**3SUD E**	0.093	0.082	0.041	**0.000**	0.044	0.017	0.063	0.069	0.357	0.082	0.093	0.355	0.063	0.339	0.345	0.397	0.081	0.395
**3SUD H**	0.098	0.050	0.032	0.060	0.058	0.051	0.088	0.031	0.388	0.050	0.098	0.392	0.067	0.377	0.379	0.428	0.102	0.426
**3SUD J**	0.075	0.059	0.033	0.040	0.026	0.040	0.050	0.060	0.354	0.059	0.075	0.357	0.063	0.342	0.345	0.394	0.076	0.392
**3SUD L**	0.064	0.059	0.039	0.044	0.033	0.042	0.041	0.071	0.346	0.059	0.064	0.355	0.058	0.339	0.340	0.389	0.062	0.387
**3SUE E**	0.085	0.090	0.054	0.036	0.029	0.040	0.055	0.082	0.337	0.090	0.085	0.333	0.074	0.317	0.324	0.375	0.079	0.373
**3SUE G**	0.073	0.070	0.043	0.044	**0.000**	0.042	0.051	0.070	0.340	0.070	0.073	0.343	0.062	0.327	0.330	0.380	0.073	0.378
**3SUE I**	0.079	0.090	0.061	0.044	0.051	0.046	0.039	0.092	0.330	0.090	0.079	0.329	0.068	0.314	0.318	0.369	0.071	0.367
**3SUE K**	0.052	0.081	0.067	0.063	0.051	0.060	**0.000**	0.102	0.314	0.081	0.052	0.323	0.059	0.309	0.308	0.356	0.043	0.354
**3SUF E**	0.066	0.051	0.036	0.053	0.025	0.049	0.056	0.062	0.352	0.051	0.066	0.361	0.057	0.346	0.346	0.394	0.071	0.392
**3SUF G**	0.078	0.045	0.038	0.060	0.054	0.050	0.073	0.059	0.366	0.045	0.078	0.377	0.039	0.363	0.361	0.409	0.076	0.407
**3SUF J**	0.097	0.119	0.088	0.066	0.061	0.071	0.058	0.117	0.308	0.119	0.097	0.298	0.097	0.282	0.291	0.344	0.087	0.341
**3SUF L**	0.065	0.076	0.051	0.043	0.028	0.043	0.030	0.083	0.328	0.076	0.065	0.331	0.057	0.316	0.318	0.368	0.061	0.366
**3SUG B**	0.070	0.062	0.057	0.063	0.062	0.055	0.059	0.088	0.340	0.062	0.070	0.355	**0.000**	0.341	0.336	0.385	0.049	0.383
**6C2M F**	0.102	0.034	0.033	0.071	0.066	0.062	0.093	0.026	0.394	0.034	0.102	0.401	0.073	0.386	0.387	0.435	0.105	0.433
**6C2M H**	0.087	0.045	**0.000**	0.041	0.043	0.031	0.067	0.038	0.372	0.045	0.087	0.375	0.057	0.360	0.363	0.413	0.084	0.411

**6C2M K**	0.089	0.036	0.012	0.052	0.049	0.042	0.073	0.034	0.377	0.036	0.089	0.383	0.059	0.368	0.370	0.419	0.088	0.416
**6C2M M**	0.082	**0.000**	0.045	0.082	0.070	0.072	0.081	0.057	0.378	**0.000**	0.082	0.394	0.062	0.380	0.376	0.422	0.087	0.420
**6P6Q C**	0.101	0.048	0.021	0.054	0.056	0.046	0.085	0.017	0.390	0.048	0.101	0.392	0.072	0.377	0.381	0.430	0.103	0.428
**6P6Q J**	0.114	0.057	0.038	0.069	0.070	0.062	0.102	**0.000**	0.405	0.057	0.114	0.406	0.088	0.390	0.395	0.444	0.120	0.442

Table S16. RMSD value for all non-hydrogen atoms of the proline ring (Figure S9) of grazoprevir conformations found by target-bound X-ray crystallography. All RMSD values are below 0.120 Å.

	**3SUD E**	**3SUD H**	**3SUD J**	**3SUD L**	**3SUE E**	**3SUE G**	**3SUE I**	**3SUE K**	**3SUF E**	**3SUF G**	**3SUF J**	**3SUF L**	**3SUG B**	**6C2M F**	**6C2M H**	**6C2M K**	**6C2M M**	**6P6Q C**	**6P6Q J**
**3SUD E**	0.000																		
**3SUD H**	0.060	0.000																	
**3SUD J**	0.040	0.054	0.000																
**3SUD L**	0.044	0.060	0.024	0.000															
**3SUE E**	0.036	0.070	0.042	0.040	0.000														
**3SUE G**	0.044	0.058	0.026	0.033	0.029	0.000													
**3SUE I**	0.044	0.081	0.047	0.045	0.041	0.051	0.000												
**3SUE K**	0.063	0.088	0.050	0.041	0.054	0.051	0.038	0.000											
**3SUF E**	0.053	0.047	0.030	0.027	0.047	0.025	0.064	0.056	0.000										
**3SUF G**	0.060	0.033	0.055	0.052	0.069	0.054	0.077	0.073	0.039	0.000									
**3SUF J**	0.066	0.110	0.066	0.069	0.048	0.061	0.045	0.058	0.083	0.106	0.000								
**3SUF L**	0.043	0.070	0.030	0.032	0.033	0.028	0.028	0.030	0.042	0.062	0.048	0.000							
**3SUG B**	0.063	0.067	0.063	0.058	0.074	0.062	0.068	0.059	0.057	0.039	0.097	0.057	0.000						
**6C2M F**	0.071	0.032	0.056	0.065	0.084	0.066	0.091	0.093	0.053	0.047	0.117	0.079	0.073	0.000					

**6C2M H**	0.041	0.032	0.033	0.039	0.054	0.043	0.061	0.067	0.036	0.038	0.088	0.051	0.057	0.033	0.000				
**6C2M K**	0.052	0.030	0.038	0.046	0.065	0.049	0.070	0.073	0.040	0.038	0.097	0.059	0.059	0.022	0.012	0.000			
**6C2M M**	0.082	0.050	0.059	0.059	0.090	0.070	0.090	0.081	0.051	0.045	0.119	0.076	0.062	0.034	0.045	0.036	0.000		
**6P6Q C**	0.054	0.024	0.046	0.056	0.068	0.056	0.077	0.085	0.048	0.046	0.104	0.068	0.072	0.022	0.021	0.018	0.048	0.000	
**6P6Q J**	0.069	0.031	0.060	0.071	0.082	0.070	0.092	0.102	0.062	0.059	0.117	0.083	0.088	0.026	0.038	0.034	0.057	0.017	0.000

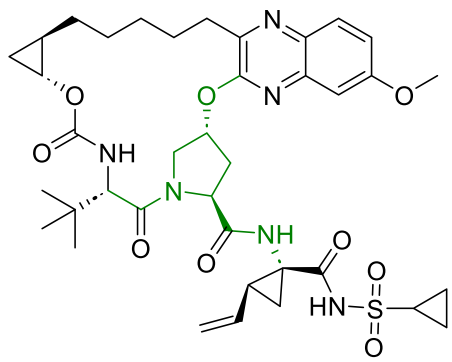

Figure S10. Structure of grazoprevir with atoms used for RMSD comparison highlighted in green.

Table S17. RMSD value for all non-hydrogen atoms of the extended proline ring (Figure S10) of the grazoprevir conformations found by MicroED, NMR, and target-bound X-ray crystallography. RMSD values of 0.285 Å or below are highlighted in green.

	**MicroED**	**DMSO-d_6 1**	**DMSO-d_6 2**	**DMSO-d_6 3**	**DMSO-d_6 4**	**DMSO-d_6 5**	**DMSO-d_6 6**	**DMSO-d_6 7**	**DMSO-d_6 8**	**CDCl3 1**	**CDCl3 9**	**CDCl3 10**	**CDCl3 11**	**CDCl3 12**	**CDCl3 13**	**CDCl3 14**	**CDCl3 15**	**CDCl3 16**
**MicroED**	0.000																	
**DMSO-d_6 1**	0.189	0.000																
**DMSO-d_6 2**	0.223	0.123	0.000															
**DMSO-d_6 3**	0.210	0.206	0.145	0.000														
**DMSO-d_6 4**	0.219	0.194	0.149	0.080	0.000													
**DMSO-d_6 5**	0.656	0.561	0.532	0.554	0.523	0.000												
**DMSO-d_6 6**	0.135	0.184	0.211	0.163	0.157	0.581	0.000											
**DMSO-d_6 7**	0.288	0.187	0.110	0.189	0.188	0.534	0.289	0.000										
**DMSO-d_6 8**	1.073	1.134	1.134	1.064	1.046	0.994	1.010	1.179	0.000									
**CDCl3 1**	0.189	**0.000**	0.123	0.206	0.194	0.561	0.184	0.187	1.134	0.000								
**CDCl3 9**	**0.000**	0.189	0.223	0.210	0.219	0.656	0.135	0.288	1.073	0.189	0.000							
**CDCl3 10**	0.999	1.055	1.047	0.989	0.968	0.894	0.933	1.097	0.361	1.055	0.999	0.000						

**CDCl3 11**	0.133	0.160	0.182	0.152	0.164	0.600	0.092	0.256	1.050	0.160	0.133	0.979	0.000					
**CDCl3 12**	0.964	1.054	1.070	1.015	1.012	1.230	0.930	1.140	0.931	1.054	0.964	0.838	0.953	0.000				
**CDCl3 13**	0.959	1.056	1.057	0.975	0.973	1.115	0.917	1.115	0.447	1.056	0.959	0.502	0.956	0.761	0.000			
**CDCl3 14**	1.052	1.156	1.166	1.086	1.081	1.238	1.017	1.226	0.492	1.156	1.052	0.590	1.059	0.785	0.178	0.000		
**CDCl3 15**	0.466	0.435	0.409	0.401	0.379	0.402	0.421	0.402	0.967	0.435	0.466	0.912	0.417	1.126	1.046	1.158	0.000	
**CDCl3 16**	1.046	1.149	1.158	1.079	1.073	1.226	1.009	1.218	0.490	1.149	1.046	0.575	1.052	0.773	0.164	0.033	1.153	0.000
**3SUD E**	0.210	0.206	0.145	**0.000**	0.080	0.554	0.163	0.189	1.064	0.206	0.210	0.989	0.152	1.015	0.975	1.086	0.401	1.079
**3SUD H**	0.260	0.174	0.122	0.136	0.117	0.524	0.230	0.109	1.127	0.174	0.260	1.059	0.203	1.096	1.066	1.174	0.378	1.168
**3SUD J**	0.193	0.168	0.133	0.081	0.080	0.534	0.122	0.207	1.055	0.168	0.193	0.974	0.124	0.985	0.971	1.080	0.405	1.072
**3SUD L**	0.177	0.159	0.146	0.106	0.080	0.528	0.101	0.216	1.041	0.159	0.177	0.964	0.118	0.991	0.967	1.073	0.385	1.065
**3SUE E**	0.237	0.209	0.151	0.094	0.058	0.503	0.164	0.206	1.040	0.209	0.237	0.959	0.180	1.008	0.971	1.081	0.383	1.073
**3SUE G**	0.219	0.194	0.149	0.080	**0.000**	0.523	0.157	0.188	1.046	0.194	0.219	0.968	0.164	1.012	0.973	1.081	0.379	1.073
**3SUE I**	0.170	0.183	0.172	0.108	0.118	0.558	0.081	0.247	1.021	0.183	0.170	0.937	0.096	0.944	0.929	1.037	0.409	1.028
**3SUE K**	0.135	0.184	0.211	0.163	0.157	0.581	**0.000**	0.289	1.010	0.184	0.135	0.933	0.092	0.930	0.917	1.017	0.421	1.009
**3SUF E**	0.199	0.175	0.144	0.091	0.062	0.557	0.155	0.187	1.074	0.175	0.199	1.007	0.147	1.016	0.990	1.095	0.403	1.088
**3SUF G**	0.223	0.155	0.114	0.124	0.099	0.536	0.195	0.134	1.104	0.155	0.223	1.037	0.164	1.063	1.038	1.144	0.372	1.138
**3SUF J**	0.223	0.260	0.211	0.107	0.124	0.560	0.154	0.265	1.006	0.260	0.223	0.920	0.186	0.952	0.909	1.019	0.414	1.010
**3SUF L**	0.189	0.166	0.164	0.117	0.093	0.529	0.109	0.224	1.031	0.166	0.189	0.946	0.138	0.981	0.949	1.055	0.408	1.046
**3SUG B**	0.133	0.160	0.182	0.152	0.164	0.600	0.092	0.256	1.050	0.160	0.133	0.980	**0.000**	0.953	0.956	1.059	0.417	1.052
**6C2M F**	0.234	0.115	0.085	0.178	0.177	0.574	0.240	0.104	1.173	0.115	0.234	1.100	0.201	1.097	1.091	1.194	0.438	1.187
**6C2M H**	0.223	0.123	**0.000**	0.145	0.149	0.532	0.211	0.110	1.134	0.123	0.223	1.047	0.182	1.070	1.057	1.166	0.409	1.158

**6C2M K**	0.222	0.091	0.056	0.167	0.166	0.532	0.205	0.139	1.144	0.091	0.222	1.055	0.181	1.066	1.065	1.172	0.434	1.163
**6C2M M**	0.189	**0.000**	0.123	0.206	0.194	0.561	0.184	0.187	1.134	**0.000**	0.189	1.055	0.160	1.054	1.056	1.156	0.435	1.149
**6P6Q C**	0.243	0.174	0.090	0.150	0.161	0.551	0.246	0.071	1.141	0.174	0.243	1.062	0.206	1.095	1.069	1.180	0.382	1.173
**6P6Q J**	0.288	0.187	0.110	0.189	0.188	0.534	0.289	**0.000**	1.179	0.187	0.288	1.097	0.256	1.140	1.115	1.226	0.402	1.219

Table S18. RMSD value for all non-hydrogen atoms of extended proline ring (Figure S10) of the grazoprevir conformations found by target-bound X-ray crystallography. RMSD values of 0.285 Å or below are highlighted in green.

	**3SUD E**	**3SUD H**	**3SUD J**	**3SUD L**	**3SUE E**	**3SUE G**	**3SUE I**	**3SUE K**	**3SUF E**	**3SUF G**	**3SUF J**	**3SUF L**	**3SUG B**	**6C2M F**	**6C2M H**	**6C2M K**	**6C2M M**	**6P6Q C**	**6P6Q J**
**3SUD E**	0.000																		
**3SUD H**	0.136	0.000																	
**3SUD J**	0.081	0.147	0.000																
**3SUD L**	0.106	0.149	0.059	0.000															
**3SUE E**	0.094	0.142	0.079	0.082	0.000														
**3SUE G**	0.080	0.117	0.080	0.080	0.058	0.000													
**3SUE I**	0.108	0.195	0.078	0.085	0.123	0.118	0.000												
**3SUE K**	0.163	0.230	0.122	0.101	0.164	0.157	0.081	0.000											
**3SUF E**	0.091	0.107	0.085	0.086	0.093	0.062	0.131	0.156	0.000										
**3SUF G**	0.124	0.060	0.125	0.119	0.125	0.099	0.166	0.195	0.081	0.000									
**3SUF J**	0.107	0.219	0.123	0.142	0.119	0.124	0.115	0.154	0.152	0.200	0.000								
**3SUF L**	0.117	0.170	0.087	0.078	0.109	0.093	0.077	0.109	0.117	0.150	0.131	0.000							
**3SUG B**	0.152	0.203	0.124	0.118	0.180	0.164	0.096	0.092	0.147	0.164	0.186	0.138	0.000						
**6C2M F**	0.178	0.116	0.172	0.182	0.196	0.177	0.210	0.240	0.152	0.117	0.254	0.194	0.201	0.000					

**6C2MH**	0.145	0.122	0.133	0.146	0.151	0.149	0.172	0.211	0.144	0.114	0.211	0.164	0.182	0.085	0.000				
**6C2MK**	0.167	0.143	0.138	0.150	0.167	0.166	0.173	0.205	0.158	0.136	0.227	0.156	0.181	0.089	0.056	0.000			
**6C2MM**	0.206	0.174	0.168	0.159	0.209	0.194	0.183	0.184	0.175	0.155	0.260	0.166	0.160	0.115	0.123	0.091	0.000		
**6P6QC**	0.150	0.107	0.173	0.181	0.181	0.161	0.204	0.246	0.159	0.111	0.227	0.198	0.206	0.107	0.090	0.134	0.174	0.000	
**6P6QJ**	0.190	0.109	0.207	0.216	0.206	0.188	0.247	0.289	0.187	0.135	0.265	0.224	0.256	0.104	0.110	0.139	0.187	0.071	0.000

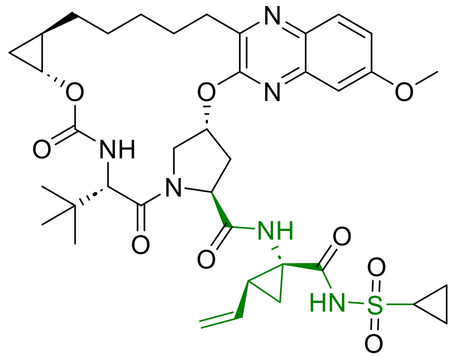

Figure S11. Structure of grazoprevir with atoms used for RMSD comparison highlighted in green.

Table S19. RMSD value for all non-hydrogen atoms of the side-chain (Figure S11) of the grazoprevir conformations found by MicroED, NMR, and target-bound X-ray crystallography. RMSD values of 0.300 Å or below are highlighted in green.

	**MicroED**	**DMSO -*d*$_6$ 1**	**DMSO -*d*$_6$ 2**	**DMSO -*d*$_6$ 3**	**DMSO -*d*$_6$ 4**	**DMSO -*d*$_6$ 5**	**DMSO -*d*$_6$ 6**	**DMSO -*d*$_6$ 7**	**DMSO -*d*$_6$ 8**	**CDCl3 1**	**CDCl3 9**	**CDCl3 10**	**CDCl3 11**	**CDCl3 12**	**CDCl3 13**	**CDCl3 14**	**CDCl3 15**	**CDCl3 16**
**MicroED**	0.000																	
**DMSO-*d*$_6$ 1**	0.737	0.000																
**DMSO-*d*$_6$ 2**	0.782	0.073	0.000															
**DMSO-*d*$_6$ 3**	0.716	0.226	0.255	0.000														
**DMSO-*d*$_6$ 4**	0.780	0.157	0.139	0.215	0.000													
**DMSO-*d*$_6$ 5**	0.711	1.098	1.139	1.052	1.144	0.000												
**DMSO-*d*$_6$ 6**	0.739	0.162	0.171	0.247	0.127	1.107	0.000											
**DMSO-*d*$_6$ 7**	0.737	0.104	0.095	0.279	0.174	1.105	0.190	0.000										
**DMSO-*d*$_6$ 8**	0.939	0.525	0.539	0.463	0.523	1.031	0.512	0.575	0.000									
**CDCl3 1**	0.737	**0.000**	0.073	0.226	0.157	1.098	0.162	0.104	0.525	0.000								
**CDCl3 9**	**0.000**	0.737	0.782	0.716	0.780	0.711	0.739	0.737	0.939	0.737	0.000							
**CDCl3 10**	0.856	0.732	0.767	0.668	0.747	0.910	0.719	0.803	0.505	0.732	0.856	0.000						

**CDCl3 11**	0.741	0.132	0.163	0.181	0.162	1.110	0.139	0.206	0.488	0.132	0.741	0.691	0.000					
**CDCl3 12**	0.955	0.780	0.817	0.821	0.865	0.998	0.796	0.840	0.777	0.780	0.955	0.759	0.772	0.000				
**CDCl3 13**	0.582	0.734	0.781	0.752	0.813	0.845	0.763	0.748	0.955	0.734	0.582	0.970	0.753	0.713	0.000			
**CDCl3 14**	0.646	0.987	1.026	0.921	1.019	0.434	0.991	0.997	0.885	0.987	0.646	0.823	0.983	1.057	0.944	0.000		
**CDCl3 15**	0.973	0.725	0.759	0.687	0.761	0.926	0.728	0.793	0.507	0.725	0.973	0.473	0.705	0.591	0.813	0.964	0.000	
**CDCl3 16**	0.521	0.482	0.525	0.535	0.548	1.017	0.507	0.489	0.821	0.482	0.521	0.905	0.493	0.815	0.435	0.944	0.871	0.000
**3SUD E**	0.716	0.226	0.255	**0.000**	0.215	1.052	0.247	0.279	0.462	0.226	0.716	0.668	0.181	0.821	0.752	0.921	0.687	0.535
**3SUD H**	0.746	0.159	0.142	0.234	0.132	1.121	0.160	0.136	0.556	0.159	0.746	0.792	0.171	0.870	0.793	0.995	0.805	0.526
**3SUD J**	0.779	0.154	0.167	0.201	0.158	1.141	0.179	0.218	0.503	0.154	0.779	0.727	0.117	0.773	0.766	1.017	0.723	0.515
**3SUD L**	0.721	0.160	0.186	0.286	0.195	1.092	0.118	0.187	0.532	0.160	0.721	0.726	0.157	0.759	0.725	0.988	0.725	0.471
**3SUE E**	0.757	0.178	0.163	0.263	0.102	1.136	0.120	0.184	0.560	0.178	0.757	0.757	0.188	0.861	0.784	1.028	0.762	0.520
**3SUE G**	0.780	0.157	0.139	0.215	**0.000**	1.144	0.127	0.174	0.523	0.157	0.780	0.747	0.162	0.865	0.813	1.019	0.760	0.548
**3SUE I**	0.712	0.197	0.218	0.162	0.181	1.100	0.170	0.243	0.519	0.197	0.712	0.720	0.147	0.780	0.726	0.979	0.731	0.483
**3SUE K**	0.739	0.162	0.171	0.247	0.127	1.107	**0.000**	0.190	0.512	0.162	0.739	0.719	0.139	0.796	0.763	0.991	0.728	0.507
**3SUF E**	0.728	0.369	0.402	0.265	0.396	0.985	0.405	0.419	0.520	0.369	0.728	0.686	0.352	0.699	0.625	0.934	0.589	0.557
**3SUF G**	0.754	0.160	0.161	0.191	0.127	1.141	0.171	0.198	0.534	0.160	0.754	0.738	0.135	0.864	0.777	1.017	0.749	0.502
**3SUF J**	0.757	0.217	0.202	0.336	0.169	1.152	0.194	0.185	0.629	0.217	0.757	0.842	0.258	0.888	0.784	1.037	0.844	0.500
**3SUF L**	0.776	0.205	0.201	0.268	0.151	1.169	0.206	0.231	0.605	0.205	0.776	0.798	0.206	0.870	0.795	1.041	0.808	0.511
**3SUG B**	0.741	0.132	0.163	0.181	0.162	1.110	0.139	0.206	0.488	0.132	0.741	0.691	**0.000**	0.772	0.753	0.983	0.705	0.493
**6C2M F**	0.733	0.462	0.488	0.487	0.523	0.906	0.499	0.477	0.661	0.462	0.733	0.779	0.499	0.664	0.520	0.970	0.580	0.587
**6C2M H**	0.782	0.073	**0.000**	0.255	0.139	1.139	0.171	0.095	0.539	0.073	0.782	0.767	0.163	0.817	0.781	1.026	0.759	0.525

**6C2M K**	0.724	0.172	0.183	0.218	0.201	1.111	0.259	0.191	0.595	0.172	0.724	0.791	0.220	0.884	0.733	0.990	0.782	0.465
**6C2M M**	0.737	**0.000**	0.073	0.226	0.157	1.098	0.162	0.104	0.525	**0.000**	0.737	0.732	0.132	0.780	0.734	0.987	0.725	0.482
**6P6Q C**	0.790	0.128	0.108	0.220	0.163	1.117	0.192	0.139	0.473	0.128	0.790	0.744	0.177	0.819	0.798	0.997	0.735	0.565
**6P6Q J**	0.737	0.104	0.095	0.279	0.174	1.105	0.190	**0.000**	0.575	0.104	0.737	0.803	0.206	0.840	0.748	0.997	0.793	0.489

Table S20. RMSD value for all non-hydrogen atoms of the side-chain (Figure S11) of the grazoprevir conformations found by target-bound X-ray crystallography. RMSD values of 0.300 Å or below are highlighted in green.

	**3SUD E**	**3SUD H**	**3SUD J**	**3SUD L**	**3SUE E**	**3SUE G**	**3SUE I**	**3SUE K**	**3SUF E**	**3SUF G**	**3SUF J**	**3SUF L**	**3SUG B**	**6C2M F**	**6C2M H**	**6C2M K**	**6C2M M**	**6P6Q C**	**6P6Q J**
**3SUD E**	0.000																		
**3SUD H**	0.234	0.000																	
**3SUD J**	0.201	0.198	0.000																
**3SUD L**	0.286	0.180	0.202	0.000															
**3SUE E**	0.263	0.139	0.214	0.169	0.000														
**3SUE G**	0.215	0.132	0.158	0.195	0.102	0.000													
**3SUE I**	0.162	0.196	0.157	0.217	0.206	0.181	0.000												
**3SUE K**	0.247	0.160	0.179	0.118	0.120	0.127	0.170	0.000											
**3SUF E**	0.265	0.417	0.332	0.426	0.430	0.396	0.310	0.405	0.000										
**3SUF G**	0.191	0.151	0.174	0.207	0.130	0.127	0.181	0.171	0.382	0.000									
**3SUF J**	0.336	0.186	0.233	0.206	0.169	0.169	0.273	0.194	0.478	0.234	0.000								
**3SUF L**	0.268	0.207	0.157	0.246	0.193	0.151	0.229	0.206	0.414	0.188	0.159	0.000							
**3SUG B**	0.181	0.171	0.117	0.157	0.188	0.162	0.147	0.139	0.352	0.135	0.258	0.206	0.000						
**6C2M F**	0.487	0.512	0.511	0.484	0.501	0.523	0.490	0.499	0.350	0.513	0.552	0.563	0.499	0.000					

**6C2M H**	0.255	0.142	0.168	0.186	0.163	0.139	0.218	0.171	0.402	0.161	0.202	0.201	0.163	0.488	0.000				
**6C2M K**	0.218	0.213	0.215	0.287	0.228	0.201	0.239	0.259	0.363	0.161	0.253	0.193	0.220	0.496	0.183	0.000			
**6C2M M**	0.226	0.159	0.154	0.160	0.178	0.157	0.197	0.162	0.369	0.160	0.217	0.205	0.132	0.462	0.073	0.172	0.000		
**6P6Q C**	0.220	0.156	0.188	0.221	0.206	0.163	0.208	0.192	0.370	0.188	0.263	0.264	0.177	0.481	0.108	0.222	0.128	0.000	
**6P6Q J**	0.279	0.136	0.218	0.187	0.184	0.174	0.243	0.190	0.419	0.198	0.185	0.231	0.206	0.477	0.095	0.191	0.104	0.139	0.000

Solubility and Cell permeability

The thermodynamic aqueous solubility, LogD and cell permeability across MCDCK cell monolayers were determined as reported previously and are presented in Table S26[1].

Table S21. MCDCK permeability data and efflux ratios (ERs) for grazoprevir.

**Solubility (μM)[a]**	**$LogD_{7.4}$[b]**	**P_{app}AB ($x10^{-6}$ cm/s)[c]**	**ER[d]**
12.9 ± 2.6	3.4	5.9 ± 0.89	3.1

[a] Determined in aqueous potassium phosphate buffer at pH 7.4. Values are means ± std from three repeats.
[b] Determined using a miniaturized shake-flask procedure. Values are means from three repeats.
[c] P_{app} AB: permeability in the apical-to-basolateral (AB) direction across MCDCK cell monolayers. Values are means + std from three repeats.
[d] ER: efflux ratio (P_{app} BA/P_{app} AB).

Literature data

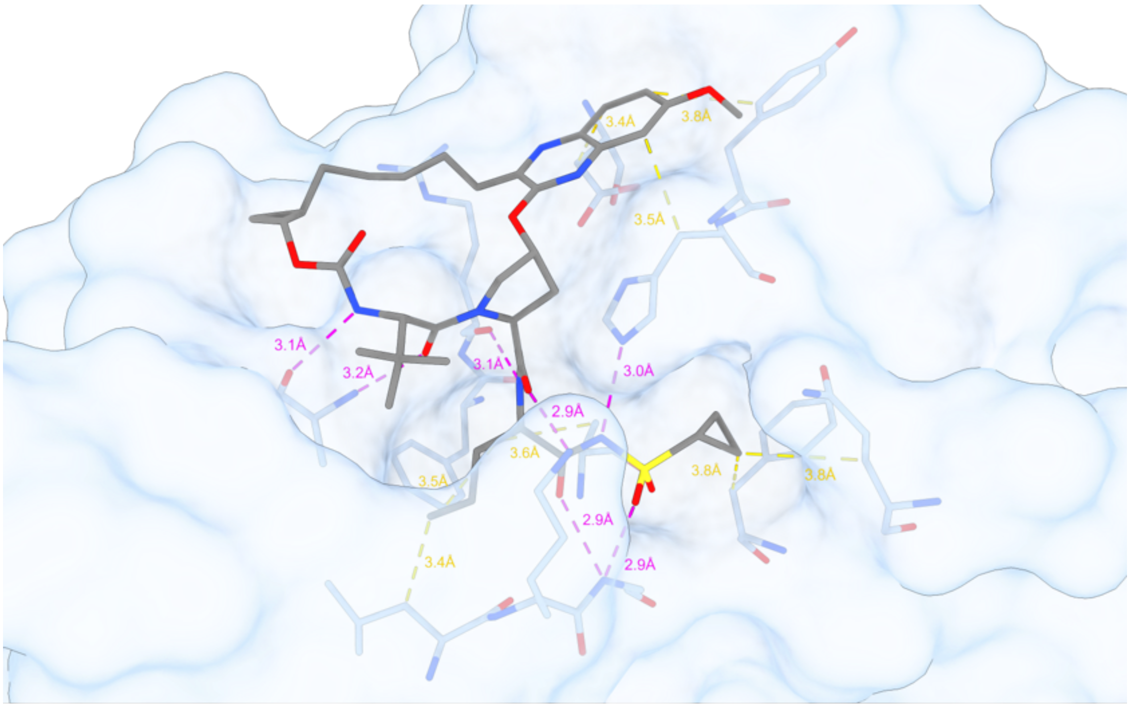

Figure S12. The binding site of grazoprevir[6]. Key intermolecular hydrogen bonds between grazoprevir and the protease are found with residues H57, K136, G137, S139A, R155 and A157. Hydrogen bonds and hydrophobic interactions are marked in magenta and yellow dashed lines, respectively.

Chameleonicity

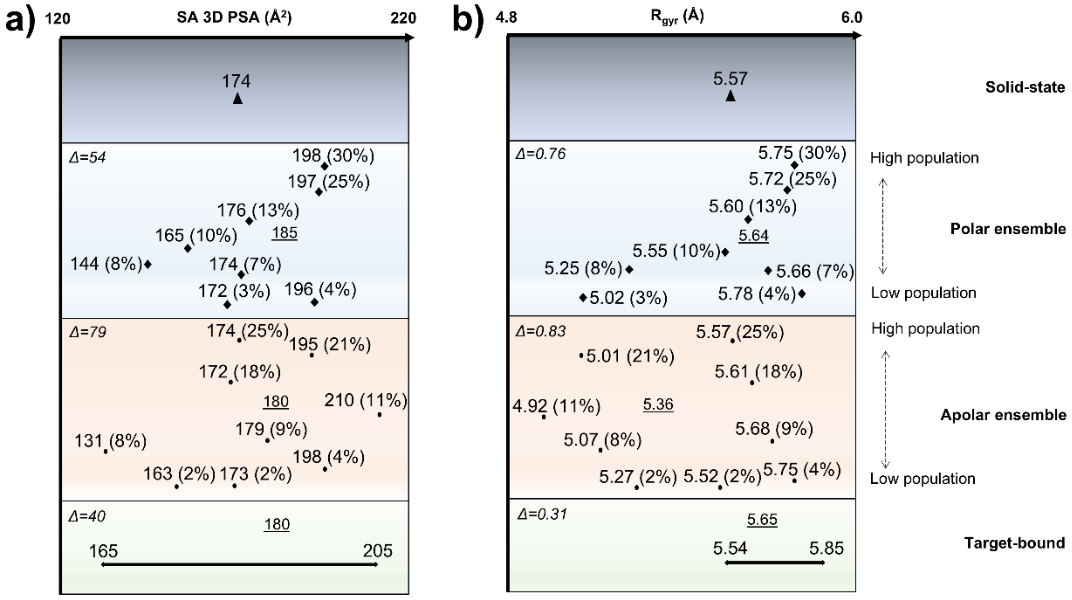

Figure S13. The calculated **a)** solvent accessible 3D polar surface area (SA 3D PSA) and **b)** radius of gyration (R_{gyr}) plotted for each conformation in the solid-state conformation, polar ensemble, apolar ensemble, and target-bound conformations (from top to bottom). In solution ensembles, the conformations are distributed along the y-axis according to their populations with the populations of each conformer given in parenthesis. In target-bound states, only the maximal and minimal values are shown. The population-weighted averaged for the solution ensembles and the averages for the target-bound conformations underscored. The deviations in each state are shown in the delta "Δ" values.

References

[1] M. Rossi Sebastiano, B. C. Doak, M. Backlund, V. Poongavanam, B. Over, G. Ermondi, G. Caron, P. Matsson, J. Kihlberg, *J. Med. Chem.* **2018**, *61*, 4189.

[2] E. Danelius, V. Poongavanam, S. Peintner, L. H. E. Wieske, M. Erdélyi, J. Kihlberg, *Chem. – Eur. J.* **2020**, *26*, 5231.

[3] L. H. E. Wieske, Y. Atilaw, V. Poongavanam, M. Erdélyi, J. Kihlberg, *Chem. – Eur. J.* **2023**, *29*, e202202798.

[4] A. Pedretti, L. Villa, G. Vistoli, *J. Mol. Graph. Model.* **2002**, *21*, 47.

[5] A. Pedretti, A. Mazzolari, S. Gervasoni, L. Fumagalli, G. Vistoli, *Bioinformatics* **2021**, *37*, 1174.

[6] K. P. Romano, A. Ali, C. Aydin, D. Soumana, A. Özen, L. M. Deveau, C. Silver, H. Cao, A. Newton, C. J. Petropoulos, W. Huang, C. A. Schiffer, *PLoS Pathog.* **2012**, *8*, e1002832.
